# Comprehensive Review of the Latest Investigations of the Health-Enhancing Effects of Selected Properties of *Arthrospira* and *Spirulina* Microalgae on Skin

**DOI:** 10.3390/ph17101321

**Published:** 2024-10-03

**Authors:** Mirosława Chwil, Rok Mihelič, Renata Matraszek-Gawron, Paulina Terlecka, Michał M. Skoczylas, Karol Terlecki

**Affiliations:** 1 Department of Botany and Plant Physiology, University of Life Sciences in Lublin, Akademicka 15 Street, 20-950 Lublin, Poland; 2 Department of Agronomy, University of Ljubljana, Jamnikarjeva 101 Street, 1000 Ljubljana, Slovenia; rok.mihelic@bf.uni-lj.si; 3 Department of Endocrinology, Diabetology and Metabolic Diseases, Medical University of Lublin, Jaczewskiego 8 Street, 20-090 Lublin, Poland; paulina.chwil@gmail.com; 4 Department of Basic Medical Sciences, The John Paul II Catholic University of Lublin, Konstantynów 1 H Street, 20-708 Lublin, Poland; emes@e-post.pl; 5 Department of Vascular Surgery and Angiology, Medical University of Lublin, Solidarności 8 Street, 20-841 Lublin, Poland; karol.terlecki@umlub.pl

**Keywords:** spirulina and phytotherapy, antibacterial activity, antifungal activity, antiviral activity, anti-acne effect, exfoliating effect, wound healing, photoprotection, skin aging, chemical profile and contraindications

## Abstract

*Arthospira platensis* and *Spirulina platensis* microalgae are a rich source of pro-health metabolites (% d.m.): proteins (50.0–71.3/46.0–63.0), carbohydrates (16.0–20.0/12.0–17.0), fats (0.9–14.2/6.4–14.3), polyphenolic compounds and phenols (7.3–33.2/7.8–44.5 and 4.2/0.3 mg GAE/g), and flavonoids (1.9/0.2 QUE/g) used in pharmaceutical and cosmetic formulations. This review summarises the research on the chemical profile, therapeutic effects in dermatological problems, application of *Arthrospira* and *Spirulina* microalgae, and contraindications to their use. The pro-health properties of these microalgae were analysed based on the relevant literature from 2019 to 2024. The antiviral mechanism of microalgal activity involves the inhibition of viral replication and enhancement of immunity. The anti-acne activity is attributed to alkaloids, alkanes, phenols, alkenes, phycocyanins, phthalates, tannins, carboxylic and phthalic acids, saponins, and steroids. The antibacterial activity generally depends on the components and structure of the bacterial cell wall. Their healing effect results from the inhibition of inflammatory and apoptotic processes, reduction of pro-inflammatory cytokines, stimulation of angiogenesis, and proliferation of fibroblasts and keratinocytes. The photoprotective action is regulated by amino acids, phlorotannins, carotenoids, mycosporins, and polyphenols inhibiting the production of tyrosinase, pro-inflammatory cytokines, and free oxygen radicals in fibroblasts and the stimulation of collagen production. Microalgae are promising molecular ingredients in innovative formulations of parapharmaceuticals and cosmetics used in the prophylaxis and therapy of dermatological problems. This review shows the application of spirulina-based commercial skin-care products as well as the safety and contraindications of spirulina use. Furthermore, the main directions for future studies of the pro-health suitability of microalgae exerting multidirectional effects on human skin are presented.

## 1. Introduction

Currently, natural raw materials with pro-health properties are being investigated for use in various pharmaceutical and cosmetic formulations; hence, there has been increased interest in the health-enhancing effects of microalgal active compounds used as components of nutraceutical and phytotherapeutic products applied in therapies of various diseases, including skin problems [1,2,3,4,5,6]. The rich chemical composition of microalgal biomass exerts a wide range of biological effects, e.g., in skin care, and attracts the attention of many manufacturers developing new innovative natural products [3,7].

Spirulina is the trade name of dried biomass of green microalgae of the genus *Arthrospira*, family Microcoleaceae, order Oscillatoriales, class Cyanophyceae, and phylum Cyanophyta [8]. It is obtained from many species of the genus *Arthrospira*, e.g., *A. fusiformis*, *A. indica*, *A. maxima*, and *A. platensis* [9,10]. *Arthrospira platensis* is one of the most popular microalgal species [11]. These microscopic cyanobacteria are characterized by a spiral or helical structure of thread-like fibres, i.e., filaments. They are three-dimensional helical microstructures with a cell wall composed of complex sugars and proteins; however, the helical transformation proceeds after mature trichomes divide into short cells chains of ~5–25 cells called hormogonia, formed and released from a larger parental trichome in a process of asexual reproduction by binary fission of the body into two new bodies, and undergo elongation. The representatives of the genus *Arthrospira* contain a peptidoglycan-polysaccharide complex in the cell wall and exhibit single thylakoid arrangement, which includes phycobilisomes on the surface [12]. The dark green unbranched threads are spiral, narrow, and either single or intertwined with each other. The length of *Spirulina platensis* cells is greater than their width; apical cells are round with a length of 7–10 microns and a width of 4–6 microns [13,14].

The systematics of the microalgae of the genus *Arthrospira* has been a taxonomic problem for many years. In 1829, Turpin classified individual *Arthrospira* species into the genus *Spirulina*, and Stizenberger established the genus *Arthrospira* in 1852. Subsequently, Castenholz separated these genera and defined their distinctive features in 1989 [15]. The term spirulina refers to a commercial product available on the market. It is also used in original research publications, which makes it difficult to introduce the correct nomenclature mainly for *Spirulina* cyanobacteria, because representatives of the genus *Arthrospira* were included in the genus *Spirulina* in the old systematics. In the current taxonomy, the genera *Arthrospira* and *Spirulina* are regarded to be related but distinct [16,17].

Due to its high nutritional value, spirulina is a component of diet and functional food [6,18,19,20]. As reported by Singh et al. [21], one gram of spirulina protein corresponds to the amount of protein contained in one kilogram of various vegetables. Microalgae exhibit multidirectional pharmacological activity: antiviral [22,23,24,25], antibacterial [26,27,28,29], antifungal [27,29], anti-biofilm [3,30,31], anti-inflammatory [32,33], antioxidant [34,35,36,37], photoprotective [3,38,39], anti-aging [11,40,41], and anti-acne [28,42,43] effects. Furthermore, microalgal compounds accelerate wound healing [44,45], alleviate herpes symptoms [3,46], and have a beneficial effect on the microbiome and the general condition of the skin [3]. 

**Figure 1:**
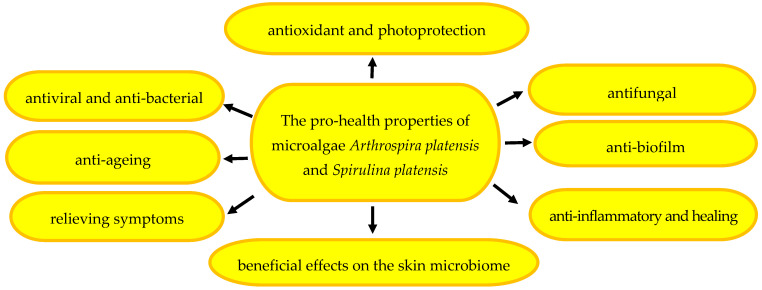
Pro-health properties of *Arthrospira platensis* and *Spirulina platensis*.

The therapeutic potential of spirulina has been confirmed in the treatment of many diseases, e.g., cardiovascular diseases [1], hypertension [47], diabetes [48], non-alcoholic fatty liver disease [49], Alzheimer’s disease [50], and infectious diseases [51], such as COVID-19 infections [2,52]. Spirulina has also been used in the chemotherapy of malignant tumours [53,54], psoriasis [55,56], acne [42,57], and oral mucositis [58,59]. Given its wide range of action, spirulina has been used for the prophylaxis and phytotherapy of dermatological conditions and as an ingredient of beauty and skin-care products [3,60,61]. The wide range of the pro-health effects of spirulina is presented in Figure 1.

The present study was undertaken to address the lack of a comparative analysis of the current knowledge of the health-promoting effects of spirulina on the skin. Therefore, an attempt was made to present issues related to the use of microalgae in therapies of dermatological problems. The aim of the study was to present the (i) chemical profile and (ii) antiviral, (iii) antibacterial, (iv) anti-acne, (v) photoprotective, and (vi) healing properties of spirulina used in the treatment of dermatological problems and to discuss the (vii) contraindications and (viii) applications of *Arthrospira* and *Spirulina* microalgae.

## 2. Results

Due to their wide range of health-enhancing properties, microalgae have been used in food, pharmaceutical, and cosmetic industries [21,62,63,64,65,66,67]. Spirulina has been used as an ingredient of pharmaceutical and cosmetic formulations and nutritional supplements. It has been tested in various biological models to determine its potential to be used as a phytotherapeutic product in such skin problems as acne [3,42,57], psoriasis [55,56], eczema [68], atopic dermatitis [3], and *molluscum contagiosum* [24].

### 2.1. Chemical Profile of Microalgae

Spirulina is characterised by a rich composition of primary and secondary metabolites and is a plentiful source of protein (60.0–76.7% d.w.) [48,69,70,71,72]. *S. platensis* microalgae contain all exogenous amino acids essential for humans: isoleucine, leucine, lysine, methionine, phenylalanine, threonine, tryptophan, and valine [73,74] as well as antioxidant barrier enzymes involved in scavenging reactive oxygen species, i.e., superoxide dismutase (SOD), catalase (CAT), glutathione peroxidase (GPx), and reduced glutathione (GSH) [35].

The polysaccharide content in *S. platensis* algae ranges from 15 to 20%, with such sugars as glucose (97%), rhamnose (2.7%), and mannose (0.4%) [70,75]. A heteropolysaccharide named SP90-1 present in these algae and composed of rhamnose, glucose, galactose, glucuronic acid, fructose, and xylose exhibits immunostimulatory and anticancer effects and stimulates the phagocytic ability and secretion of nitric oxide (NO), interleukin (IL)-1β, IL-6, and tumour necrosis factor α (TNF-α) in RAW264.7 cells. Moreover, it inhibits the growth of A549 lung cancer cells [70].

The crude fat content in *A. platensis* spirulina is in the range of 1.5–2.5%, which is lower than in *Chlorella vulgaris* (5.7%) and *Azolla pinnata* (4.0%) [69,76]. While the group of unsaturated fatty acids in *S. platensis* is dominated by γ-linolenic acid (GLA, C18:3 n-6, 39%) and α-linolenic acid (ALA, C18:3 n-3; 14%) characterised by high antioxidant activity [77], the main fatty acids in *A. fusiformis* hot extract are palmitic acid (PA, C16:0; 55.2%) and stearic acid (STA, 18:0; 27.1%) [29]. Alpha-linolenic acid (ALA, C18:3n-3), eicosapentaenoic acid (EPA, C20:5n-3), and stearidonic acid (SDA, 18:4 n-3) are the dominant omega 3-fatty acids, whereas mainly γ-linolenic acid (GLA; C18:3 n-6), arachidonic acid (ARA, C20:4 n-6), and linoleic acid (LA, C18:2 n-6) represent omega 6-fatty acids [73,74,77,78]. Fatty acids have promising applications in pharmaceutical and cosmetic preparations [29].

The content of polyphenolic compounds in the methanol extract of spirulina is in the range of 7.8–44.5 mg GAE/g. The dominant polyphenols are benzophenone > propanediamine > phenylacridine > piperidine > carbanilic acid > pyrrolidine > oxazolidin-2-one > dinitrobenzoate [79]. Spirulina contains high amounts of phenols (0.3 GAE/g d.w.) and flavonoids (0.2 mg quercetin/g d.w.), with the dominance of kaempferol, quercetin, and apigenin. The group of phenolic acids is dominated (mg/kg) by succinic acid (1123), quinic acid (844), and 3,4-hydroxybenzoic acid (687). In turn, chlorogenic, ferulic, caffeic, coumaric, and syringic acids occur in lower amounts [69,77]. The high concentrations of phenolic compounds and fatty acids are responsible for the antioxidant properties of microalgal biomass [77].

The results of investigations of the proximate composition of primary and secondary phytochemical compounds and their metabolites present in *Arthospira platensis* were recently presented by [76]. The proximate composition of spirulina powder contains crude protein (58.9%), crude fat (1.5%), crude fibre (1.0%), ash (12.2%), moisture (9.6%), carbohydrates—total nitrogen-free extract (16.7%), gross energy (4183 kcal/kg), and sand and silica—acid insoluble ash (0.31%), and mineral salts of calcium (0.3%), phosphorus (1.1%), and total salt (1.2%). Spirulina contains higher levels of crude protein (58.9%) than *Chlorella vulgaris* (47.1%) and *Azolla pinnata* (21.8%). In turn, the crude fat and fibre levels, total salt, and calcium, as well as sand and silica in spirulina (1.5, 1.0, 1.2, 0.3, and 0.3%, respectively), are lower than in chlorella (5.7, 2.9, 1.6, 0.8, and 0.8%) or azolla (4.0, 26.2, 4.1, 2.3, and 0.6%). The moisture content, carbohydrates, and gross energy in spirulina (9.6%, 16.7%, and 4183 kcal/kg, respectively) are lower than in chlorella (9.9%, 26.7%, and 4377 kcal/kg) and higher than in azolla (9.3%, 15.8%, and 3295 kcal/kg). Moreover, spirulina is characterized by a higher content of ash and phosphorus (12.2 and 1.1%, respectively) than chlorella (7.8 and 1.0%) and lower levels than azolla (22.8 and 1.2%) [76]. Qualitative detection of secondary phytochemicals in the methanolic extract of spirulina showed a high content of polyphenols and tannins and moderate levels of saponins, quinines, and cardiac glycosides. In turn, petroleum ether extracts of these microalgae revealed high levels of alkaloids, moderate levels of flavonoids and terpenoids, and a low content of tannins and polyphenol. The petroleum ether extracts showed the absence of saponins, cardiac glycosides, and quinones, while no alkaloids, terpenoids, and flavonoids were detected in methanolic extracts. Therefore, the maximum quantity of tannins and phenols was found in methanolic extracts, whereas the maximum amount of flavonoids was found in petroleum ether extracts. The values of total flavonoids, tannins, and phenols were (mg/g d.w.) 1.4, 6.9, and 5.9, respectively, in spirulina methanol extracts and 4.8, 3.0, and 2.7, respectively, in petroleum ether extracts [76].

Gas Chromatography-Mass Spectrometry (GC-MS) analysis of *A. platensis* showed the presence of 11 secondary metabolic compounds, with six detected in methanolic extracts and five in petroleum ether extracts. Bioactive secondary phytochemical metabolic compounds detected in spirulina petroleum ether extracts were represented by docosane (C_22_H_46_) and 9-octadecenal (oleic acid, C_18_H_34_O), while methanolic extracts showed the presence of tetradecanoic acid (myristic acid, C_14_H_28_O_2_) and hexadecanoic acid (palmitic acid, C_16_H_32_O_2_). The other secondary metabolic compounds identified in petroleum ether extracts of these microalgae included 5-iodo-5-(1′-naphthyl)-1-phenoxypent-4-en-2-ol (C_21_H_19_IO_2_), 2-propanone (C_3_H_6_O) and pentadecanoic acid, 14-methyl-, methyl ester (C_17_H_34_O_2_), while methanolic extracts showed the presence of formic acid, butyl ester (C_5_H_10_O_2_), 2-bromolauric acid (C_12_H_23_BrO_2_), cyclohexane, 1,3,5-trimethyl-2-octadecyl-(C_27_H_54_), and N-ethyl-N-methyl-4-nitrosobenzenamine (C_9_H_12_N_2_O). Collectively, 12 principal bioactive compounds—hexadecanoic acid; 9-octadecenal; docosane; tetradecanoic acid; dodecanoic acid; phenol, 2-methoxy-5-(1-propenyl)-, (E); 6,7-dimethoxy-2-tetralone; 4-(6,6-dimethyl-2-methylenecyclohex-3-enylidene) pentan-2-ol; 3,7,11,15-tetramethyl-2-hexadecen-1-ol; cycloheptane, 1,3,5-tris(methylene); cis-2-[2-(hydroxymethyl)cyclopentyl] ethanol, and loliolide—were found in *A. platensis* [76].

A comparative analysis of some chemical properties of powdered spirulina (*Spirulina platensis*) samples from the USA, China, Belgium, and Bulgaria was carried out, with determination of the content of elements, tocopherols, and fatty acids. As shown by the results, the total lipid content in the samples varied from 1.9% (China) to 6.1% (Belgium). Spirulina from Bulgaria, unlike the samples from the other countries, contained more monounsaturated fatty acids (MUFAs) than polyunsaturated fatty acids (PUFAs), and the highest content of unsaturated fatty acids was determined in samples originating from the USA. The samples of spirulina from Belgium and China contained approximately 15% of ω-6 fatty acids, and the American samples had less than 4% of these acids, while they were not found in the Bulgarian spirulina, which confirms that cyanobacteria are a source of ω-6 but not ω-3 fatty acids. Palmitic acid (C16:0) was the main saturated fatty acid in the samples from all of four countries. In the Chinese samples, palmitic acid was followed consecutively by linoleic (C18:2), oleic (C18:1), stearic (C18:0), and palmitoleic acids (C16:1), while the fatty acid distribution in the American samples was as follows: palmitic > oleic > palmitoleic > stearic ≈ linoleic acid. In addition, the Belgian samples of spirulina showed a level of 100% of α-tocopherol, while no tocopherols were detected in the Bulgarian samples, but these samples showed the highest Ba (4.2 mg/kg) and Ca (5132 mg/kg) content and the lowest values of Sr (25.3 mg/kg) and S (4833 mg/kg). In turn, the highest Sr (141 mg/kg) and S (5833 mg/kg) values were recorded in the samples from Belgium, and Chinese spirulina was characterised by the lowest Ca content (1483 mg/kg). The Cr content in the samples derived from the USA (3.4 mg/kg) exceeded those recorded in Bulgaria (2.5 mg/kg), Belgium (2.5 mg/kg), and China (2.5 mg/kg) [80].

Various concentrations of photosynthetic pigments in spirulina have been reported. The chlorophyll content in dry, freeze-dried, and frozen *S. platensis* samples ranges from 1925 to 9080 µg·g^−1^ d.w. [81]. The percentage of chlorophyll and c-phycocyanin is 1.2 and 17.2%, respectively [82]. The carotenoid content in spirulina is 235 mg·g^−1^ [83] (these compounds were represented by beta- and alpha-carotene, zeaxanthin, astaxanthin, echinenone, cryptoxanthin, xanthophyll, and lutein) [82,84,85]. Additionally, photosynthetic pigments, i.e., chromatophores of phycobiliproteins, such as phycocyanin, phycoerythrin, phycobilisomes, porphyrin, and tetrapyrrole, were detected in these algae [86,87].

The group of minerals in spirulina is dominated by iron (7.6–31.6%), selenium (0.01–39%), calcium (0.2–30%), phosphorus (3–27%), and potassium (0.5–7%), whereas chrome, copper, magnesium, manganese, sodium, and zinc are detected in lower concentrations [73]. Furthermore, spirulina is a valuable source of vitamins A, E, C, D, K, and group B vitamins (B1, B2, B3, B5, B6, B7, B8, B9, and B12) [25,35,82,88,89].

Bioactive chemical compounds contained in spirulina modulate signalling pathways in metabolic processes and regulate the expression of proteins and the activity of enzymes, e.g., superoxide dismutase [90,91]. Currently, many studies focus on the use of various spirulina fractions: lipids [3,92,93], polysaccharides [94,95], peptides [96,97,98,99], pigments [12,100,101,102], and phenolic compounds [103,104,105] in dermatology and cosmetology. Spirulina is an alternative source of natural antioxidants and other metabolites that may be useful in the phytotherapy of some skin diseases [77]. 

It has recently been discovered that a phycobiliprotein-derived bioactive peptide, IRDLDYY, named SpirPep1, from *Spirulina* (*Arthrospira plantensis*) strain C1, due to the presence of the hydrophobic amino-acid residues of the peptide, is a natural inhibitor of the Angiotensin-I-Converting Enzyme (ACE) with IC_50_ of 1.748 mM and is non-toxic to fibroblasts from African green monkey kidney and human dermal skin [106]. Another two novel ACE inhibitory peptides from *Spirulina platensis* were obtained with amino acid sequences Val–Thr–Tyr (VTY) and Leu–Gly–Val–Pro (LGVP) and IC_50_ values of 23.39 µM and 45.76 µM, respectively [107].

### 2.2. Spirulina for the Needs of Persons with Skin Diseases

Skin aging is mainly caused by mechanical damage, genetically determined hormonal changes, UV radiation, active or passive smoking, quality of nutrition, skin care, stress, diseases, and sleep deprivation. With age, the ability of keratinocytes to proliferate and the number of Langerhans cells, melanocytes, fibroblasts, macrophages, and intercellular junctions decrease, and enzymatic degradation of hyaluronic acid, collagen, and elastin takes place. The regenerative capacity of the skin is reduced. The lower sebum secretion leads to skin dryness and changes in the composition of the hydrolipid barrier, making the skin more susceptible to environmental factors, including microorganisms [108,109]. Additionally, the protective barrier can be weakened by many diseases, e.g., diabetes and demodicosis, which is common in the elderly, or by unfavourable living conditions, e.g., working in water or wearing a face mask, even in young or middle-aged subjects [110,111,112,113,114].

There are many substances, including those of natural origin, e.g., antimicrobial peptides, which can be used as ingredients of cosmetics to limit the presence of microorganisms on the skin surface [115,116,117]. In turn, the multidirectional action of various biologically active chemical compounds from *A. platensis* and *S. platensis* microalgae applied topically on the skin or orally includes their anticancer [4,115,118,119,120], antioxidant [4,121,122,123,124], and anti-aging [40,125,126] properties.

### 2.3. Pro-Health Effects of Microalgae on Skin

#### 2.3.1. Antiviral Effect

**Figure 2:**
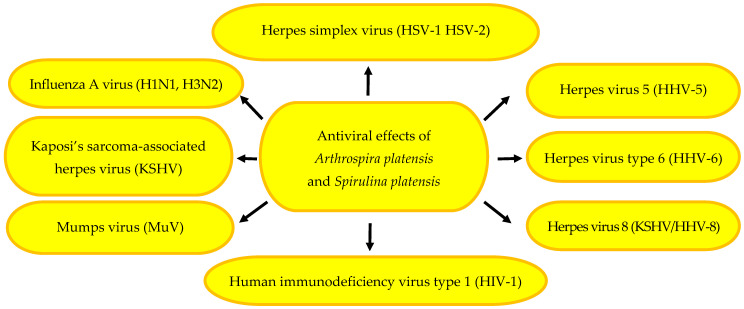
Antiviral effects of *A. platensis* and *S. platensis*.

The antiviral activity of microalgae is associated with their rich profile of active metabolites, i.e., proteins, lectins, polysaccharides, and photosynthetic pigments. Some active compounds have been used in genetic engineering for recombination of nucleic acids and production of new vaccines, e.g., against SARS-CoV-2 [127,128]. As a source of sulphated polysaccharides, polyphenols, and lectins with antiviral and immunomodulatory properties, microalgae are a promising strategy in the fight against viruses. The mechanism of their action is based on the inhibition of viral replication and enhancement of human resistance to viral infections [23]. Polysaccharides, calcium spirulan, a sulphated polysaccharide composed of rhamnose, 3-O-methyl-rhamnose, 2,3-di-O-methyl-rhamnose, and 3-O-methylxylose, uronic acids, sulphate groups, and calcium ions chelated with sulphate groups isolated from *S. platensis* and other microalgae increase the resistance of the organism to infection [23,46]. They inhibit the replication of herpes simplex virus (HSV-1, HSV-2), cytomegalovirus (HCMV), herpes virus 5 (HHV-5), herpes virus 8 (KSHV/HHV-8), measles virus (MeV), mumps virus (MuV), herpes virus type 6 (HHV-6), influenza A virus, Kaposi’s sarcoma-associated herpes virus, and HIV-1 virus [22,23,46]. Phycobiliproteins, primarily, c-phycocyanin and allophycocyanin, inhibit infections caused by influenza A and B viruses. The mechanism of their action involves blocking the replication of the viruses and their entry into host cells [23]. The antiviral effects of *A. platensis* and *S. platensis* is presented in Figure 2.

The potential of *Spirulina platensis* to mitigate adverse metabolic syndrome effects (oxidative stress, inflammation, and mitochondrial functional disruption) results from the highly active antiretroviral therapy (HAART) in HIV treatment. These corrective health properties of spirulina are mainly attributed to antioxidant pigments: chlorophyll, carotenoids (β-carotene), and phycocyanin [129]. Chlorophyll has antioxidant and antimutagenic activities. Spirulina is a rich source of chlorophyll *a*; however, this chlorophyll form is believed to be less effective than chlorophyll *b* in antioxidant activity. In turn, the activity of chlorophyllin is even more effective than that of β-carotene, retinol, vitamin C, and vitamin E [130,131,132]. β-Carotene is commonly known for its anti-carcinogenic, antioxidant, and anti-inflammatory activity. This membrane antioxidant protects against singlet oxygen (^1^O_2_)-mediated lipid peroxidation. β-carotene is a suppressor of inflammatory mediators, including nitrogen monoxide (NO), prostaglandin E(2), inducible nitric oxide synthase (iNOS), tumour necrosis factor α (TNF-α), interleukin-1 beta (IL-1β), and interleukin (IL)-6/IL-12 family cytokines, and this activity results from the ability to inhibit the nuclear factor kappa B (NF-κB) activation via prevention of nuclear translocation of the transcription factor p65, also known as the NF-κB p65 subunit [133]. 

Phycocyanin, similar to β-carotene, inhibits the TNF-α formation and COX-2 expression and decreases prostaglandin E(2) production. It decreases iNOS expression, scavenges alkoxy, hydroxyl, and peroxyl radicals, and reduces oxidative stress and the level of nicotinamide adenine dinucleotide phosphate (NADPH) oxidase (NOX). Moreover, phycocyanin inhibits liver microsomal lipid peroxidation and prevents the degradation of cytosolic inhibitor of NFκB (IκB-α). It has been revealed that the inhibitory activity of phycocyanin results from the suppression of TNF-α formation in macrophages. Phycocyanin regulates mitogen-activated protein kinase (MAPK) activation pathways, i.e., the p38 and c-Jun N-terminal kinase (JNK), and exerts a regulatory effect on extracellular-signal-regulated kinase (ERK1/2) pathways [129].

*Spirulina platensis* enhances the activation and expression of endothelial nitric oxide synthase (eNOS) and heme oxygenase 1 (HO-1). The latter is an enzyme that probably plays a crucial role in the adaptive reprogramming and activation of nuclear factor erythroid 2-related factor 2 (Nrf-2) and, in consequence, the production and increased expression of catalase (CAT) and superoxide dismutase (SOD). Moreover, spirulina activates the Nrf2/HO-1 pathway [134]. It has been shown that hot water extracts of *Arthrospira maxima* (spirulina) have anti-respiratory syncytial virus (RSV) activity, and the polysaccharide-enriched high-molecular weight fraction (>100 kDa, SHD1), characterised by high rhamnose content and 3-4- and 2,3-Rhap linkages as the main glycosyl linkages, significantly reduces viral yield and may be recommended as an effective candidate for development of novel drug against RSV infection. SHD1 disrupted RSV internalisation and inhibited binding of RSV attachment (G) protein to heparan sulphate receptors on the host cell surface, thus preventing RSV attachment and entry. SHD1 showed a half maximal effective concentration (EC_50_) of 0.0915 mg/mL and a selective index (SI) of >261.5 against RSV [135].

The high content of proteins and vitamins in *A. platensis* biomass contributes to its activity against HIV, HSV, and SARS-CoV viruses, antioxidant properties, and ability to strengthen the immune system [22]. The health-enhancing effectiveness of calcium spirulan against the HSV-1 virus is based on the chelation of calcium ions with sulphate groups, which prevents infection caused by adsorption and penetration of the virus into host cells [46]. The effectiveness of *S. platensis* against this virus is also influenced by a rhamnose-containing polysaccharide and a glycolipid (sulfoquinosyl diacylglycerol) with IC50 values of 21.3 and 6.8 ng/mL, respectively. In turn, *S. maxima* metabolites reduce HSV-2 infection in the initial stages of virus adsorption and penetration (ED50; 0.07 mg/mL) [25]. The potency of inhibition of Mayaro virus replication by terpenoids and unsaturated aliphatic particles from various microalgal species, including *A. maxima*, was found to be higher than that of the antiviral drug ribavirin [136]. 

Spirulina exopolysaccharides have antiviral properties and block viral infection by binding the virus to the cell surface, thereby preventing its spread. This mechanism of action involves metabolic pathways with anti-inflammatory, immunomodulatory, and antioxidant properties. Exopolysaccharides can be regarded as a promising component of pharmacological, cosmetic, and functional food products [137]. Some scientific reports have shown that the ethanol-extracted exopolysaccharide compound (0.5%, 1%, and 5%) from *Spirulina* spp. can be made into a fine and stable lotion formula. The odourless white lotion with soft structure and pH from 5.75 to 6.15 did not show the presence of solid particles in the homogeneity test. The spreadability of the lotion evaluated in a dispersion test was in the range of 5.8–6.0. Additionally, the exopolysaccharide lotion did not cause irritation [138].

A cream formulation with a defined microalgal extract (Spiralin^®^) and a 1.5% sulphated polysaccharide named calcium spirulan, which successfully prevented *herpes labialis* in a trial with susceptible individuals, are proposed as novel cosmeceutical treatment options for children with *molluscum contagiosum*—a common viral skin infection affecting children. The results of an open-label observational study showed that Spirularin^®^ VS cream applied two times a day over a period from 1 to 9 months (mean treatment duration about 4 months) revealed that 73.1% of patients achieved complete clearance of active lesions [24].

Some microalgal metabolites are alternative therapeutic agents in infections caused by enveloped viruses (*Herpes simplex virus, measles virus, and mumps virus*) and non-enveloped viruses (astrovirus and rotavirus). *S. platensis* and *S. maxima* extracts inhibit the spread of these viruses in host cells. Moreover, spirulina stimulates the human immune system, which suggests that it may be a potential therapeutic supplement [128]. Microalgae are beneficial sources of new antiviral drugs and parapharmaceuticals used in the treatment of various viral infections [137]. Additionally, a diet enriched with microalgae has antiviral properties [127].

#### 2.3.2. Antibacterial and Antifungal Activity

The interest in microalgae as safe ingredients of pharmaceutical and cosmetic products with antibacterial and antioxidant properties is constantly growing [29]. The new trend involves the search for natural microalgal pigments, and their application is associated with their impact on the skin microflora. The content of phycocyanins in *A. platensis* extracts is estimated at 38%, and these compounds regulate the growth of *Staphylococcus* and *Klebsiella*. They also modify some bacterial species or the entire microflora on the skin surface [126]. Phycocyanins isolated from *S. platensis* inhibit the growth of antibiotic-resistant bacteria, e.g., *Escherichia coli*, *Klebsiella pneumoniae*, *Pseudomonas aeruginosa*, and *Staphylococcus aureus* strains [25].

A study of the antimicrobial activity of phycobiliproteins isolated from *A. fusiformis* against 13 species of bacteria and fungi, e.g., *Aspergillus flavus*, *A. niger*, *Candida albicans*, *Escherichia coli*, *Proteus vulgaris*, *Salmonella typhi*, *S. typhimurium*, and *Serratia marcescens*, showed the susceptibility of eight microbial species to these compounds. Given their antibacterial and nutritional properties, the active compounds isolated from *A. fusiformis* may serve as potential therapeutic ingredients of human skin-care and therapy products [29]. Moreover, microalgal active metabolites inhibit various species of the genus Vibrio effectively [25]. Methanol, hexane, and combined extracts of *S. platensis* contain natural potentially bioactive compounds with high antibacterial activity and can be used to develop a safe natural agent against multidrug-resistant bacteria found in commercial hair and scalp skin cosmetics as well as face-care, foot-care, and body-care products. These extracts applied at 0.5 mg/mL were highly active against four pathogenic isolates with high resistance to antibiotics, which were genetically identified as *Bacillus cereus*, *Staphylococcus* sp., *Pseudomonas aeruginosa*, and *Stenotrophomonas maltophilia*, with an inhibition zone ranging between 2 mm and 23 mm [139]. 

*Spirulina platensis* may be successfully used as a safe and environmentally friendly anti-bacterial compound in soap. The best recognised NaOH concentration as alkali to use in such soap with this microalga is 3.75 M with 0.478% of free fatty acid and 14.03% of water. Spirulina contains fatty acids which are necessary in soap making, including myristic acid (0.01–0.03 g/100 g), palmitic acid (2.00–2.50 g/100 g), stearic acid (0.01–0.05 g/100 g), oleic acid (0.10–0.20 g/100 g), linoleic acid (0.75–1.20 g/100 g), and γ-linoleic acid (1.00–1.50 g/100 g) [140]. Spirulina has been used as a source of fatty acids to prepare antibacterial soap in reaction with NaOH characterised by activity against *Staphylococcus aureus* (MRSA). The hot process method at a temperature of 65 °C with the use of 1 g *Spirulina platensis* as the optimum composition in soap making was employed [141].

Among three fractions of biopeptides from A. maxima OF15 obtained through enzymatic hydrolysis with subtilisin A (PHA), pepsin hydrolysis (PHP), and hydrolysis with both these enzymes (PHS), only PHP showed promising potential of bactericidal action against all four human pathogenic bacteria examined in the experiment (*Escherichia coli*, *Salmonella typhi*, *Bacillus subtilis*, and *Staphylococcus aureus*), displaying the MIC (minimum inhibitory concentration) value of 1.25 (mg mL^−1^) against the first two Gram-negative bacteria and 0.63 (mg mL^−1^) against the other two Gram-positive bacteria with IC_50_ of 0.94, 0.99, 0.34, and 0.62 (mg mL^−1^), respectively. PHP exhibited the highest efficiency against E. coli [142]. Bioactive molecules of *Spirulina platensis* exhibited similar activity to that of a standard synthetic antifungal agent (Amphotericin B) against *Aspergillus niger* (CTM 10099) and *Alternaria alternata* (CTM 10230), and in particular, against the *Fusarium* genus: *Fusarium oxysporum* (CTM10402), *Fusarium culmorum* (ISPAVE 21w), and *Fusarium graminearum* (ISPAVE 271). The inhibition zone diameters (mm), minimal inhibitory concentrations (MIC), and minimal fungicidal concentrations (MFC) of spirulina against pathogenic fungi were as follows: 15.75, 0.156, and 0.625 (*F. oxysporum*), 15.00, 0.156, and 0.625 (F. *culmorum*), 15.25, 0.156, and 1.250 (*F. graminearum*), 11.25, 0.625, and 2.5 (*A. niger*), and 10.25, 0.156, and 0.312 (*A. alternata*). Promising results were also obtained with the use of a *Spirulina* and pomegranate peel combination [143].

Studies of the biological and pharmaceutical efficacy of ethanol and alkaloid extracts of *Spirulina platensis* against dermatophytes isolated from patients at the Imam Hussein Teaching Hospital revealed that these extracts did not exert a protective effect against *Trichophyton rubrum* at a concentration of 50 mg, but they were efficient at 100 mg, with increased activity at 200 mg. Moreover, both these spirulina extracts were effective against *Trichophyton concentricum* and *Trichophyton interdigitale*, and their activity was enhanced with the increasing concentration [13].

#### 2.3.3. Alleviation of Acne Symptoms

Bacterial infections are one of the causes of acne. Acne therapy is based on antibiotic- containing products, e.g., clindamycin and erythromycin. Long-term application of these drugs leads to development of antibiotic resistance in bacteria. Therefore, natural antibacterial agents for acne therapy inducing no side effects are being sought [28,43]. One of the natural methods of this therapy is the use of bioactive spirulina compounds with deep skin penetration abilities [43]. Hexane and methanol extracts of *S. platensis* exert antibacterial effects against various Gram-positive (*Aerococcus* spp., *Enterococcus* spp., *Staphylococcus aureus*, and *Staphylococcus epidermidis*) and Gram-negative (*Escherichia coli*, *Pseudomonas aeruginosa*, and *Pseudomonas stutzeri*) bacteria isolated from acne skin lesions. The antibacterial activity is mainly attributed to the presence of hexadecene, heptadecane, 2-bromopropionic acid, benzenedicarboxylic acid, methyl-1-docosene, octadecene, and tetradecanol. These compounds can be used to develop natural antibiotics against resistant bacteria for acne therapy [42]. Moreover, microalgal active compounds have a beneficial effect on the condition and appearance of the skin [3]. Ethyl acetate extracts from *S. platensis* applied at concentrations of 20,000 and 30,000 ppm exhibited potent antibacterial activity against *Enterobacter aerogenes* (inhibition zone 11.7 and 12.6 mm, respectively), *Propionibacterium acne* (inhibition zone 14.4 and 16.9 mm, respectively), and *Staphylococcus epidermidis* (inhibition zone 13.1 and 13.2 mm, respectively). Gram-positive bacteria were more sensitive to the extract than Gram-negative bacteria. The antibacterial activity was influenced by the structure and components of the bacterial cell wall. The extract fractions were dominated by bis(2-ethylhexyl) phthalate, 1,2-benzenedicarboxylic acid, and bis(2-ethylhexyl) [28].

Topical ointment formulated with the use of C-phycocyanin (C-PC) extracted from spirulina using sonication and cold-maceration and further purified with the dialysis method showed quite good antimicrobial action and was effective against *Propionibacterium acne* and *Staphylococcus epidermidis* in the treatment of acne. The minimum inhibitory concentration (MIC) of the first developed formulation comprising a water-soluble base (aqueous extract of spirulina) against both these microorganisms was 1.5 mg/mL and 1.8 mg/mL, respectively, and the diameter of the inhibition zone was 26.1 mm and 24.6 mm. The second formulation, prepared using an oleaginous base, showed a MIC value of 1.6 mg/mL and 2.1 mg/mL against *P. acne* and *S. epidermidis*, respectively, and an inhibition zone of 23.1 mm and 21.3 mm. Therefore, the water-based formulation is more effective in inhibiting bacterial proliferation than the oil-based one. Both formulations had good consistency. The water-base formulation had a globule diameter of 5.44 mm, pH of 6.8, viscosity of 175 cps, and spreadability of 8.6 g cm/s, while the values of these parameters recorded for the oil-base preparation were 5.29 mm, 6.1, 198 cps, and 8.1 g cm/s, respectively. The oil-base formulation had a lower spreadability (8.1 g cm/s) than the water-base one (8.6 g cm/s) [144]. However, other studies did not demonstrate antibacterial activity of ethanol extracts of *Spirulina platensis* obtained from the Indonesian region against Staphylococcus aureus and Escherichia coli [145].

Phycocyanins isolated from *S. platensis* exhibited antimicrobial, anti-inflammatory, and antioxidant properties. These compounds can be used as a natural safe ingredient of hydrophilic anti-acne ointments and creams for topical use in acne and other skin diseases [146,147]. Due to the presence of alkaloids, steroids, saponins, and phenol, a face mask containing *S. platensis* ethanolic extract inhibited *Cutibacterium* acnes with an inhibition zone diameter of 10 mm, comparable to that observed for the synthetic antibacterial drug—clindamycin. Therefore, microalgae may be a beneficial antibacterial ingredient in cosmetics recommended for acne-prone skin [60,148]. Among various cream formulations with the addition of *S. platensis* and non-ionic surfactants (Polysorbate 60, Cremophor A6:A25 (CR) (1:1), Tefose 63, and sucrose ester SP 70) with Transcutol HP, a formulation containing the sucrose ester SP 70 emulsifying agent had the strongest antimicrobial activity against *Cutibacterium* acnes and *Staphylococcus* aureus and the highest antioxidant activity against UVB-induced oxidative stress on HaCaT. This formulation exhibited the finest dissolution profiles and low toxicity. Therefore, *Spirulina platensis* cream may be successfully used topically in acne therapy with fewer side effects and without antibiotic resistance [57].

It was demonstrated that Ce6 trimethylester from *S. platensis* cultivated in Vietnam is a promising potential photosensitizer in photodynamic antibacterial therapy for the treatment of acne. Ce6 trimethylester with halogen light exerted a strong antimicrobial effect against skin bacteria *Propionibacterium acnes* VTCC 0218 and *Staphylococcus aureus* VTCC 0173 with a MIC99 value of 1.25 μg/mL. Such therapy was also effective in the treatment of cancer cells, as the cell survival and colony formation rates (proliferation and cologenicity) of HeLa cells declined as the Ce6 trimethylester treatment concentration increased [149].

Bioactive compounds extracted from *S. platensis* and nanoparticles of water-soluble chitosan, i.e., an active nanocarrier of chemical compounds, were used to carry out the encapsulation process. Chitosan, which is a natural cationic polymer, binds organic compounds characterised by chelating, biocompatible, antibacterial, and biodegradable properties [43,150]. Hydrogel (spirulina-chitosan) induces the proliferation of HFF1 (Human Fibroblast) cells to a great extent and upregulates the transforming growth factor beta (*TGF-β*) and Platelet-Derived Growth Factors (*PDGF*) gene expressions promoting wound healing; hence, it is a potential cosmeceutical and biomedical product [151,152]. To enhance the penetration of active compounds through the skin, chitosan nanoparticles and ethanol extracts of spirulina containing saponins, tannins, steroids, and phenols were used at a concentration of 3.25, 7.5, and 15 mg/mL in the encapsulation process. This complex exhibited antibacterial activity against *Staphylococcus aureus* and *Pseudomonas aeruginosa* (inhibition zone 5–10 mm), which confirmed that the nanoencapsulation process increased the absorption of the extract by bacterial cells. Spirulina-chitosan nanoparticles with deep skin penetration potential can serve as antibacterial agents in acne therapy and can be used in cosmeceutical products [43]. The pro-health properties of microalgae determined in vitro studies are shown in Table 1.

#### 2.3.4. Exfoliating Effects

Ethanol extracts of *Spirulina platensis* (2%) were successfully used for the preparation of an exfoliating peel-off gel mask with polyvinyl alcohol (PVA) and hydroxypropyl methylcellulose as a gelling agent (DMC). Three formulas (A, B, and C) of this cosmetic with different PVA and DMC ratios were prepared. The percentage ratio of PVA to DMC was 2.25:1.25 in formula A, 2.00:1.05 in formula B, and 1.50:1.15 in formula C. Formulas A and B met all the requirements of physical stability tests, i.e., for formulation A, a pH of 5.00, dispersion of 10.30 cm, viscosity of 557 CP, and time to dry of 29 min 21 s; and for formulation B, a pH of 5.00, dispersion of 11.20 cm, viscosity of 207 CP, and time to dry of 32 min 01 s [154]. The proteolytic activity of *Arthrospira platensis* lysate, confirmed by kinetics analysis and zymography with different substrates, pH values, and divalent ions, which revealed the presence of two proteolytic enzymes, was successfully used to develop an enzyme-based cosmeceutical hydrogel formulation with potential application as a topical exfoliating agent. The incorporation of spirulina extracts into this hydrogel formulation markedly improved its operational stability over time [155].

There are a few scientific reports showing that spirulina helped reduce psoriasis in mice, improving the symptoms of eczema. It was found that *Spirulina maxima*-derived C-phycocyanin treatment (1 μg, 2 μg, and 5 μg) eliminated the presence of characteristic silver scale redness and thickness of the psoriatic skin. It also lowered epidermal thickening and immune clustering in the dermis. The epidermal thickness in the psoriasis group was 88.727 μm, while the C-phycocyanin-treated group was characterised by an epidermal thickness comparable to that observed in the positive control, i.e., the mometasone (Novastone TM cream)-treated group—31.338 μm; however, the histological differences were not significant among the C-phycocyanin treated samples. Furthermore, C-phycocyanin modulated the mRNA expression and the level of inflammatory cytokines (tumour necrosis factor-α TNF-α, interleukin (IL)-6, IL1b, cyclooxygenase (COX)-2) in a female BALB/C-nu mouse model and inhibited psoriasis-related cytokines (interferon (IFN)-γ, IL-17a, calcitonin gene-related peptide CGRP) in female BALB/c mice, which resulted in a reduced degree of redness and scaling. Therefore, C-phycocyanin may be considered as a natural pharmaceutical agent against psoriasis [55,56].

Nurmukhambetov et al. [68] proposed an innovative method for the external treatment of chronic lichenified eczema, which was more efficient than the traditional therapy based on pure celestoderm. It consisted of the inclusion of an ointment prepared by the authors and containing spirulina (*Spirulina platensis*) with anti-inflammatory and antioxidant effects into traditional therapy three times a day for two weeks. In addition to spirulina, which constituted 20% of the product by weight, the ointment contained lanolini—15.0%, vaselini—10.0, glucocorticosteroid celestoderm reducing a variety of inflammatory and toxic-allergic reactions occurring in soft tissues during chronic allergic dermatoses—15.0%, urea responsible for keratolytic and moisturizing effect—8.0%, boric acid—1.0%, zinc oxide—4.0%, and dimexide exerting an anti-inflammatory and penetrating effect—3.0%. In comparison to conventional therapy, the use of the ointment as part of the complex therapy of chronic eczema increased the effectiveness of the treatment two-fold, causing a more pronounced regression of the symptoms of the disease and reducing its severity. The use of the proposed complex therapy for the external treatment of such chronic dermatoses as lichenified eczema improved the quality of patients’ life and alleviated inflammatory phenomena: erythema of oedema and infiltration. It contributed to disappearance of excoriation, lichenification, and dry skin and to healing of cracks in lesions, thereby leading to full restoration of the normal skin structure.

#### 2.3.5. Healing Effect

Microalgae are a rich source of active chemical compounds with pro-health properties supporting tissue regeneration, accelerated granulation, and wound healing [156,157,158]. The healing effect is achieved through spirulina effects on the proliferation of fibroblasts and keratinocytes, inhibition of inflammatory (TNF-α, NF-B, TlR-4, VEGF) and apoptotic processes (AIF, caspase-3), and reduction of the expression of the HGMB-1 protein involved in the regeneration of damaged tissues, and an increase in angiogenesis and collagen fibre density [3,159,160]. Antioxidants present in spirulina inhibit the generation of intracellular reactive oxygen species (ROS) and mitigate skin cell aging [39].

Increased cell proliferation and viability in injury sites promote the granulation process in wounds [161]. Growing attention is paid to the application of natural methods based on the use of microalgal active ingredients, e.g., spirulina, alginate, or chitosan, in the treatment of wounds and prophylaxis of infections [157,158,162]. These products have antiviral, antibacterial, antifungal, antiprotozoal, and anti-inflammatory activity [163,164,165,166]. The active compounds contained in spirulina, i.e., phycocyanins, carotenoids, γ-linolenic acid (GLA, 18:3 n-6), and selenium, have antioxidant properties. Chitosan accelerates wound healing through the activation of fibroblasts and polymorphonuclear cells, production of cytokines, participation in the synthesis of type IV collagen, and migration of macrophage cells [167].

Nanophytosomes containing *S. platensis* extract (3 mg/mL) stimulate collagen production, inhibit anti-apoptotic activity, and increase the effectiveness of active compounds [159]. Other encapsulated *S. platensis* protein hydrolysates with nanoliposomes enhance the growth of the HFFF-2 epidermal fibroblast cell line. It was also found that nanoliposomal peptides accelerate wound healing through angiogenesis and collagen production [45].

Spirulina proteins accelerate the process of skin wound healing through an increase in the expression of α-smooth muscle actin (α-SMA), SOD and CAT activity, and reduction in malondialdehyde (MDA) levels in granulation tissue. They increase the phosphorylation and activation of the extracellular signal-regulated kinase (ERK) protein and the level of Smad2 protein phosphorylation. They also stimulate collagen expression in granulation tissue and accelerate wound healing. It is known that the Akt, ERK, and TGF β1 signalling pathways are involved in this process [46]. A study on male Wistar rats aged 2–3 months with skin incision revealed that the application of an ointment containing *S. platensis* extract (0.1%) reduced inflammation, increased the number of fibroblasts, and accelerated the healing process. This suggests that microalgae can be used in biomedicine and cosmetics to accelerate the healing process [168]. 

Spirulina crude protein promotes human dermal fibroblast viability (cell line CCD-986sk) via activation of the epidermal growth factor receptor (EGFR) and mitogen-activated protein kinase (MAPK)/extracellular signal-regulated kinase (ERK) signalling pathways [169]. In an in vitro human dermal fibroblast (HDF) model, it was revealed that *Spirulina maxima*-derived marine pectin (SmP 12.5–50 μg/mL) enhanced cell proliferation by 20–40%. Simultaneously, the wound-healing results in HDFs showed that spirulina at a concentration of 12.5 and 25 μg/mL decreased the open wound area by 32 and 12%, respectively, compared to the control (44%). In an in vivo model of zebrafish (*Danio rerio*) larvae, a markedly greater fin regenerated area was shown in the SmP-treated group at a concentration 50 μg/mL. In turn, in adult zebrafish, the open skin wound healing % was markedly greater after topical application (600 μg/fish) of SmP (46%) than in the control (38%). The qRT-PCR assay revealed the upregulation of tgfβ1, timp2b, mmp9, tnf-α, and il-1β genes as well as chemokines cxcl18b, ccl34a.4, and ccl34b.4 in the muscle and kidney tissues of SmP-treated fish. The rapid epidermal growth and tissue remodelling in the SmP-treated fish was further supported by histological analysis [170]. Moreover, in the zebrafish caudal fin regeneration model, reduction of neutrophils in the wound region was observed after treatment with PUFAγ-linolenic acid (GLA)-enriched glycolipids extracted from *Arthrospira* (*Spirulina*) *platensis* with the supercritical-CO_2_ method. This proves that spirulina in the GLA form possesses anti-inflammatory, anti-oxidative, and anti-allergic activities, which act in a concerted manner to promote post-injury regeneration in zebrafish [171].

A novel spirulina water-soluble polysaccharide extracted from CO_2_-enriched *Arthrospira platensis*—a hetero-polysaccharide with molecular weight 6.21 kDa composed of rhamnose, xylose, glucose, and mannose—is proposed to be an excellent source of natural wound healing and/or a cytotoxic remedy in rats. The healing effect of this semi-crystalline polysaccharide composed of 100 to 500 µm geometrically shaped units with flat surfaces may result from several mechanisms, including an increase in the rate of re-epithelialisation and neo-vascularisation, free radical scavenging, reduction of inflammation, and control of infection [124].

Investigations of the potential of *Spirulina platensis* to be used for cutaneous excisional and burn wound healing in a Wistar rat skin model revealed that spirulina maintained moisture of the wound site and promoted wound healing by enhancing wound angiogenesis and collagen deposition, weakening histopathological and morphological alterations, and inhibiting scar formation via the upregulation of angiogenic genes, such as angiogenic basic fibroblast growth factor (bFGF) and vascular endothelial growth factor (VEGF), as well as downregulation of fibrotic genes, such as transforming growth factor-β1 (TGF-β1) and α-smooth muscle actin (α-SMA). A histopathological examination showed that the topical supplementation with spirulina contributed to marked epithelisation, inflammatory cell infiltration, angiogenesis, extracellular matrix deposition, and wound contraction, i.e., remodelling of connective tissue. Therefore, the healing potential of spirulina for skin healing and its role as a promising antifibrotic agent against scar formation has been evidenced [44]. Studies on rats Wistar (*Rattus norvegicus*) showed no differences in the effectiveness of topical administration of 96% and 70% of *Spirulina platensis* ethanol extract gel towards the density of collagen fibres in the treatment of traumatic ulcers in the lower labial mucosa on the third day, while differences in the effectiveness were recorded on the seventh day [172]. Studies on the antioxidant and wound-healing activities of *Spirulina* spp. revealed that, in comparison to ethanol, acetic acid, and buffer extracts, those extracted with water for one hour with the maceration method had stronger antioxidant capacity with IC_50_ 45.21 mg/mL, AEAC 0.12 mg Vit. C/g sample, phycocyanin content 6.478 µg/mL, and chlorophyll content 2.642 µg/mL. Treatment with 1% spirulina extract had a stronger wound-healing effect on Wistar rat skin than 10% *Spirulina* spp. extract and wound-healing ointment containing povidone-iodine 10%. In addition, 1% spirulina water extract exerted a greater wound-contraction effect (62.5%) than 10% povidone-iodine (22.5%) and produced no irritation and toxicity effect in the skin of female Swiss Webster mice (20–40 g) and albino rabbits (1.5–2 kg) [122].

**Figure 3:**
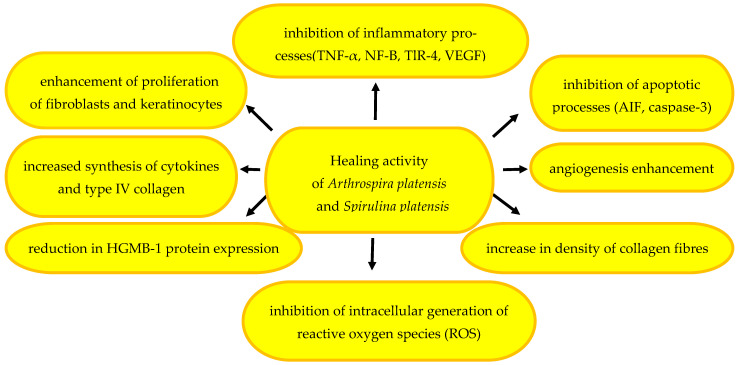
Selected mechanisms of the healing action of *A. platensis* and *S. platensis*.

There is a new trend consisting of searching for natural pigments to be used as substitutes for synthetic ones, which may be toxic. The phycocyanins from *S. platensis* included in a cream proved able to enhance the wound-healing effect, demonstrating effective inhibition of the growth of *Aspergillus fumigatus*, *A. niger*, *Candida albicans*, and *Escherichia coli* at the same time [173]. This fact, along with the findings that spirulina and chlorella supplementation accelerates angiogenesis, proliferation of epithelial cells, and formation of granulation tissue in diabetic rats, shows that these ingredients may be recommended for wound therapy in diabetic patients [174]. The active metabolites of *S. platensis* can be regarded as potential agents to be used in complementary and conventional medicine to heal various mechanical, chemical, and thermal wounds [175]. Antioxidant peptide ML11 obtained from the *A. platensis* transglutaminase core domain exerts an antioxidant effect via intracellular ROS scavenging. Nanofibres encapsulated with peptide ML11 have a positive effect on the wound-healing process. They can be used as a biomaterial or a biopharmaceutical drug restoring redox balance in adjuvant wound-healing therapy [121]. Selected mechanisms supporting the wound-healing process after the use of *A. platensis* and *S. platensis* microalgae are shown in Figure 3.

#### 2.3.6. Photoprotective Effect

Currently, there is an increasing demand for natural skin photoprotectants against ultraviolet radiation (UVB), which causes various skin problems: changes in pigmentation, sunburn, photoaging, and cancer [176]. Spirulina has photoprotective and antioxidant properties; when added to sunscreens, it removes free radicals generated as a result of radiation, thereby increasing the level of photoprotection [177].

Polyphenols contained in *S. platensis* extracts, which are rich in phenolic compounds (23,466 mg GAE/kg of extract) with a high degree of absorbance and SPF 30.4 (3 mg/mL), are involved in the photoprotective properties. They inhibit intracellular ROS generation in fibroblast cells, tyrosinase activity, and the secretion of pro-inflammatory cytokines IL-6 and IL-8. The extracts are a good source of UVB radiation-absorbing antioxidant metabolites and can be used as an ingredient of photoprotective cosmetic products [176]. Spirulina phytochemicals exert a photoprotective effect through the inhibition of tyrosinase. They also have a beneficial effect on the proliferation of skin fibroblasts and keratinocytes, the extracellular matrix, and collagen production [3]. *S. platensis* and *S. maxes* metabolites can be regarded as pro-health active ingredients of natural cosmetics for skin care, sun protection, and the acceleration of wound healing; they also exhibit moisturising, anti-wrinkle, anti-aging, and anti-acne properties [147].

The photoprotective properties of microalgae are mainly regulated by phlorotannins, carotenoids, and mycosporine-like amino acids, i.e., shinorine, porphyra, palythine, asterina, palythinol, and usujirene; they are used in formulations intended for topical application. Some metabolites isolated from microalgae are used as components of sunscreens and anti-aging, regenerating, emollient, and hair-care products [178]. Spirulina-loaded bilosomes constitute a novel anti-aging drug delivery system and a promising nanoplatform transferring microalgal metabolites. They can be used to treat UV-induced skin damage, ensuring maximum therapeutic effects [179].

Recently, the clinical profile of spirulina in skin diseases was assessed in a study conducted in a tertiary care hospital, Bangladesh. The facial gel-cream formulation containing *Spirulina* extract (0.1% *w*/*w*) applied twice daily for four weeks markedly enhanced the stratum corneum water content and reduced transepidermal water loss TEWL in both young (20–39 years old) and mature (18–65 years old) skin groups of tested healthy volunteers. The former effect was more pronounced in the mature skin group, and the latter effect was stronger in the older group than in the younger group. Additionally, the formulation containing the active ingredient substantially decreased the amount of sebum in the skin and improved the skin microrelief by reducing surface roughness (Ser parameter). Additionally, ultrasound imaging analysis showed only slight and insignificant improvement in the echogenicity of the dermis of the skin of the young and mature volunteers using the spirulina gel-cream. After four weeks of treatment with the spirulina formulation, an improvement of the skin microrelief by the reduction of the surface roughness was noted, and keratinocytes were more uniformly distributed and homogeneous without significant changes in the epidermis thickness. Therefore, spirulina extract can be regarded as a unique innovative active ingredient of effective multifunctional dermocosmetic formulations intended for the care of young and mature skin with protective anti-aging benefits. Spirulina improves skin conditions, especially epidermis structure, and provides long-term hydration, protection of the skin barrier function, and oil control. It is responsible for the maintenance of the skin hydrolipidic film balance, providing unique skin benefits with the balance of moisture and skin lipid content mainly in mature skin [60]. 

A study on the anti-aging properties of phycocyanins purified from the cyanobacterium *A. platensis* has shown that this phycobiliprotein greatly delays the chronological aging of wild-type W303-1A *Saccharomyces cerevisiae* yeast cells as a model organism used for studying aging and aging-associated diseases, and this effect was also recorded under calorie restriction (0.2% glucose) in the growth medium. The majority of the aged phycocyanin-treated cells unable to form colonies were ROS + (dihydrorhodamine 123 (DHR123) staining)/PI − (propidium iodide staining). The high longevity accompanied by the enhanced level of ROS in the phycocyanin-treated cells at the time of inoculation may be explained by the fact that phycocyanin might promote longevity by inducing and adaptive mechanism called hormesis. On the other hand, phycocyanin addition three days after inoculation, i.e., at the end of the growth phase, resulted again in enhanced anti-aging activity; however, the ROS production was lower or comparable to that in untreated cells, suggesting that, in this case, phycocyanins may favour survival through a mechanism other than hormesis, i.e., signalling mechanisms [41].

Recently, the photoprotective and skin-ageing properties of spirulina have been investigated in terms of the effective inhibition of matrix metalloproteinases (collagenase, elastase, hyaluronidase, and tyrosinase), which is the focus of many marketed cosmeceutical formulations. The inhibition of hyaluronidase, elastase, and collagenase is related to a decrease in wrinkles and enhancement of skin elasticity, but the inhibition of tyrosinase is attributed to skin whitening and anti-melanogenesis treatments. It was demonstrated that, in addition to its antioxidant, antimicrobial, and anti-inflammatory activities, *Arthrospira maxima* was able to produce anti-collagenase peptides, which have a sequence that resembles the cleavage point in native collagen and, therefore, competes with the collagenase active site, preventing extracellular collagen matrix breakdown. In comparison to the synthetic inhibitor 10-phenanthroline, three fractions of biopeptides from A. maxima OF15 obtained via enzymatic hydrolysis with subtilisin A (PHA), pepsin hydrolysis (PHP), and hydrolysis with both these enzymes (PHS) displayed superior anti-collagenase activity. 10-Phenanthroline exhibited a level of inhibition activity of 57.13 at 75 µg mL^−1^, whereas at the same concentration PHA and PHP showed values < 70 and PHS had a value of 92.5%, with IC_50_ of 96.7, 43.9, and 32.5 µg mL^−1^, respectively. The anti-inflammatory activity of the three peptide fractions was confirmed by the inhibition of hyaluronidase (Type IV). To the best of our knowledge, this is the only scientific report showing that an *Arthrospira*-derived peptide may be involved in hyaluronidase inhibition. The highest percentage value of inhibition of the hyaluronidase enzyme by PHS (38.8%), PHA (<32%), and PHP (<32%) was 333 µg mL^−1^ with IC_50_ of 0.92, 1.63, and 1.66 mg mL^−1^, respectively. This inhibitory effect on hyaluronidase activation was probably related to the presence of polysaccharides. It was also discovered that extracts, specifically ethanol-insoluble fractions of *Spirulina platensis*, may be anti-allergic substances. The genus *Arthrospira* is known for its antibacterial activity mainly due to the production of phycocyanins and carotenoids, while the antibacterial peptides of these microalgae are rarely reported [142]. In a human dermal fibroblast cell line (CCD-986sk) model, it was found that the levels of expression of both aging-associated matrix metalloproteinase-8 (MMP-8), i.e., a collagen-degrading enzyme, and elastase were significantly decreased by spirulina crude protein (6.25, 12.5, and 25 µg/mL), leading to increased collagen levels. Spirulina crude protein treatment of CCD-986sk cells reduced elastase activity, with a maximum decrease of 42% recorded in cells treated with 25 µg/mL, and promoted the secretion of procollagen type I C-peptide in a dose-dependent manner. Compared to the basal level of 77 ng/mL, procollagen type I C-peptide concentrations of 100, 121, and 187 ng/mL were induced by 6.25, 12.5, and 25 µg/mL of spirulina protein, respectively [169].

The Spirulina polysaccharide complex has been proposed as an anti-aging agent rejuvenating fibroblasts, i.e., restoring the mitochondrial function and collagen production by increasing their antioxidant potential via upregulation of superoxide dismutase 2 (SOD2), but not by activation of inflammatory pathways. Furthermore, the Spirulina polysaccharide complex stimulated endoplasmic reticulum (ER) protein folding by upregulating the expression of ER chaperones [180]. *Arthrospira platensis* crude proteins (25 μg/mL) protect fibroblasts against oxidative stress induced by H_2_O_2_ [181].

The latest scientific studies conducted on young (6–8 weeks old, weight: 120–180 g) and old (20–22 months old, weight: 250–300 g) female rats (*Rattus norvegicus*) have shown that spirulina extract delays the signs of skin aging by enhancing collagen in both intrinsic and extrinsic aging. The subacutenous injection of microalgae extract (20 mg/mL daily for a week) before UVA irradiation (2.16 J/cm^2^), in addition to the recovery of collagen density and reduction of the production of matrix metalloproteinases, exerted significant anti-ageing effects mediated by antioxidation (increase in superoxide dismutase—SOD and reduced glutathione—GSH), anti-inflammatory effects (downregulation of the expression of inflammatory cytokines: interleukin-1β IL1β and tumour necrosis factor-α TNF-α), and matrix metalloproteinase-1 (MMP-1) inhibition in chronological and photo-aged skin of albino rats. Therefore, spirulina extract is a useful component for application in dermocosmetic formulations intended for improvement of the epidermis structure and protection of the skin from the effects of aging and UV exposure [182]. Other studies have reported a strong inhibitory effect of spirulina-derived C-piroccrocin on UVB-induced secretion of matrix metalloproteins MMP-1 and MMP-9 in keratinocytes (HaCaT cells) together with enhanced secretion of involucrin, filaggrin, and loricrin as well as reduced ROS production. Hence, spirulina C-piroccrocin is proposed as a protective agent against UVB-induced damage, restoring the physical barrier function of the skin and preventing or reducing skin aging via reducing skin wrinkles and free radicals [39]. The results of studies on the bioactivity of phycoerythrin and phycocyanin extracted from *Spirulina* sp. and Nostoc sp. in a human skin fibroblast cell line (CCD-966SK) revealed that the largest percentage of collagen I was detected at 125 and 62.5 ppm, respectively, while the lowest quantities were found at 1000 ppm of both pigments. The latter concentration (1000 ppm) of the pigments was shown as the most active for malondialdehyde (MDA) concentrations, superoxide dismutase (SOD), and glutathione peroxidase (GPx) activity, and the release of anti-inflammatory IL-6 and TNF-α cytokines. The highest collagen III concentrations were noted at 3.91 and 15.63 ppm of phycoerythrin and phycocyanin, respectively. Therefore, both pigments exert beneficial effects in enhancing necrotic, anti-inflammatory, and enzymatic activity against the human skin fibroblast cell line [183].

**Figure 4:**
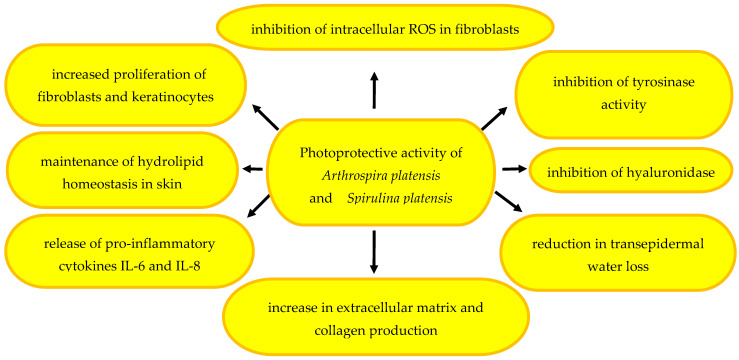
Photoprotective action of *Arthrospira platensis* and *Spirulina platensis*.

There is one scientific report demonstrating the anti-photoaging effects of an N-terminal acetylated and C-terminal amidated *Spirulina platensis*-derived hexapeptide in UVB-irradiated human immortalised keratinocytes (HaCaT cells) in 7–8-week-old female Kunming mice. The acetylated and amidated hexapeptide severely decreased the MDA content by 49%, enhanced catalase (CAT), superoxide dismutase (SOD), and glutathione peroxidase (GSH-Px) activities by 103%, 49%, and 116%, respectively, and reduced MMP-1 and MMP-3 expression by 27% and 29%, respectively. Moreover, novel isobaric tags for relative and absolute quantitation (iTRAQ)-based proteomic analysis were employed, and 60 differential proteins were identified and mapped into an interaction network with two core subnetworks, and the key metabolic pathways were determined [184]. Selected mechanisms of the photoprotective action of *A. platensis* and *S. platensis* are presented in Figure 4.

Due to the tyrosinase inhibitory activity, *Arthorpira platensis* crude extract or its main constituents acts as an anti-melanogenesis or skin-whitening agent useful for treating hyperpigmentation and is an excellent potential candidate in developing effective and safe skin-whitening cosmetics. Tyrosinase catalyses the first step in two-step reactions of melanin synthesis: the hydroxylation of l-tyrosine to 3,4–dihydroxyphenylalanine (l-dopa) and the oxidation of l-DOPA to highly reactive dopaquinone that polymerises spontaneously forming melanin. UV light enhances the tyrosinase expression and melanin synthesis within melanosomes, i.e., organelles of melanocytes. Melanosomes are transferred to surrounding keratinocytes, where melanin degradation occurs and, in consequence, the skin becomes tanned [122].

It was found that the ethanol spirulina extract exhibited stronger inhibitory activity towards tyrosinase (IC_50_: 1.4 × 10^−3^ g/mL) than the water extract (IC_50_: 7.2 × 10^−3^ g/mL), and ferulic and caffeic acids were the main components recognised as the main tyrosinase inhibitors in the ethanol extract [101]. As reported by [122], *Spirulina* spp. water extract was an effective whitening agent with inhibitory activity against tyrosinase of IC50 0.51 mg/mL. *Spirulina*-derived C-phycocyanin, especially at the dose of 0.1 mg/Ml, inhibited tyrosinase activity and reduced melanin content. Two dual anti-melanogenic mechanisms of this biliprotein were recognised: upregulation of MAPK/ERK-dependent degradation of the transcription factor of tyrosinase (MITF) and downregulation of p38 MAPK-regulated CREB (transcription factor of MITF) activation to modulate melanin formation [7].

The *Spirulina*-derived tripeptide Asp-Glu-Arg (DER) with a ‘-CDOCKER_Energy’ value of 121.26 Kcal mol^−1^ effectively inhibits tyrosinase activity. The half maximal inhibitory concentration of this peptide that binds to tyrosinase residues His85, His244, His259, and Asn260 is 1.04 mmol L^−1^. These residues are the key drivers of the interaction between the peptide and tyrosinase [185]. *Spirulina*-derived solid polyphenol extract (gallic acid) prepared by extraction of spirulina with ethyl alcohol (volume concentration of 80%) and then decompression concentration at 40 °C and freeze-drying has been recognised as a tyrosinase inhibitor [122]. To conclude, the tyrosinase inhibition by spirulina extracts confirms their potential as a skin whitening compound.

The strong inhibitory effect of C-phycocyanin contained in *Spirulina platensis* protein extracts on the activity of human gelatinases MMP-2 (by 55.13%) and MMP-9 (by 57.9%) as well as the drop in the mRNA expression of both gelatinases has been documented in the hepatocellular cancer cell line HepG2. These matrix metalloproteinases degrading basement membrane and denaturing structural collagens are essential in degrading collagen fragments after their initial degradation by [100]. The antioxidant effect is particularly important in the case of cosmetics intended for skin with signs of aging. All the three biopeptide fractions from A. maxima OF15 mentioned above exhibited high capacity, even stronger than that of Vitamin C, for sequestering the free radical 2,2-diphenyl-1-picryl-hidrazol (DPPH), but the strongest potential scavenging activity was shown for PHS. The percentage sequestering of the DPPH free radical by PHA, PHS, and PHP was 78, 78, and 77% at a concentration of 0.1 g mL^−1^ and IC_50_ values of 21.25, 34.63, and 17.93 µg mL^−1^, respectively. The strong capacity of the peptides against free radicals was confirmed in the 2,2′-azinobis-3-etilbenzothiazoline-6-sulfonic acid (ABTS) radical scavenging assay. PHS and PHA exhibited strong reducing power with Trolox^®^ Equivalent Antioxidant Capacity (TEAC) values of 540.7 and 465.7 μM of Trolox/g sample and IC_50_ of 8.6 and 9.5 µg mL^−1^, respectively, while PHP showed TEAC of 282.2 μM of Trolox/g sample and IC_50_ of 15.63 µg mL^−1^. This high total antioxidant capacity probably results from the presence of cysteine (Cys) and methionine (Met) in their structure or the aromatic side chains of the histidine (His) and tyrosine (Tyr) amino acids, which very easily donate hydrogen atoms. Studies of the iron-chelating activity of *Arthrospira*-derived peptides revealed that PHA (93% at 25 µg mL^−1^) was more efficient than PHP (<30% at 25 µg mL^−1^) and PHS (<30% at 25 µg mL^−1^) and even than the commercial chelator Na_2_-EDTA (61% at 25 µg mL^−1^) with IC_50_ of 6.97, 724.7, 492.2, and 14.31 µg mL^−1^, respectively. This strong chelating activity of PHA is probably related to the presence of methionine (MET), lysine (LYS), and arginine (ARG) [142]. The pro-health effects of some microalgae were presented in various research models animal studies and clinical trials (Table 2 and Table 3).

### 2.4. Contraindications

Spirulina is considered safe for human consumption, which has been confirmed during the long period of its use as a food source strengthening the immune system and providing energy. The World Health Organization (WHO) and the Food and Agriculture Organization (FAO) have identified spirulina as the leading food of the future, as it contains easily digestible essential nutrients [165,166,187,188]. Spirulina is classified as a superfood due to its rich nutritional value. However, side effects related to the presence of certain toxic compounds have been demonstrated during short- and long-term consumption [189].

Commercial spirulina supplements have been found to contain cyanobacterial toxins. Microcystins (MC) (36–584 ng g^−1^ d.w.) from the group of hepatoxins destabilise the central and peripheral nervous system. The cytotoxin cylindrospermopsin (CYN) (221–352 ng g^−1^ d.w.) disrupts many metabolic processes in the cell. In turn, β-methylamino-L-alanine (0.5 μg·g^−1^ d.w.), classified as a neurotoxin, irritates mucous membranes and skin [82,190,191]. When consumed every day, the concentrations of the toxins specified above pose a threat to children’s health [82,189,190,191]. Microalgal toxins can cause acute poisoning, cancer, liver damage, and gastrointestinal disorders. Long-term consumption of spirulina may initiate the development of Alzheimer’s disease and Parkinson’s disease. The safe daily recommended spirulina dose for adults is approximately 3–10 g, with 30 g/day as the maximum intake limit [82,191].

Spirulina should be excluded from the diet of subjects with an allergy to this product [192,193,194,195]. Consumption thereof is especially dangerous during pregnancy and breastfeeding, as toxins may enter the child’s organism through breast milk [195,196]. Spirulina should be excluded from the diet of patients with autoimmune diseases and those taking herbal drugs, anticoagulants, and hypoglycaemic agents to prevent interactions [196]. Moreover, it should not be used by phenylketonuria patients, as they are not able to metabolise the amino acid phenylalanine contained in spirulina [197]. It should also be eliminated from the diet of patients with blood clotting disorders, as this process is disrupted by phycocyanin, which inhibits cyclooxygenase (COX-2) [196]. Additionally, other relevant metabolic disorders, such as tyrosinaemia, should be mentioned as a contraindication to spirulina intake. 

One of the ways to eliminate hazardous substances from spirulina is ecological cultivation in accordance with accepted standards and norms [198,199]. Therefore, to protect consumers’ health, there is a need for a stringent control system with regular monitoring of the presence of toxins at all stages of spirulina production [73,189].

The safety profile of spirulina consumption has been confirmed in clinical rodent and human studies. However, there are reports on the toxicity of consumption of preparations of spirulina grown in open water sources, which may contain trace amounts of mercury and heavy metals. Spirulina supplements have very rarely caused adverse reactions in humans. There have been isolated cases of rhabdomyolysis (muscle breakdown) and the mixed immunoblistering disorder with characteristic features of bullous pemphigoid, i.e., a rare skin condition with blisters on the skin, and pemphigus foliaceus, which is an autoimmune skin disease representing bullous dermatoses characterised by erosive-desquamative lesions, short-term blisters, and a chronic course [200].

### 2.5. Application in Industry

The pharmacological properties of microalgae have been used in nutraceutical and cosmetic preparations for treatment of dermatological problems [3,201]. Due to its therapeutic activity, i.e., immune system strengthening, photoprotective, anti-aging, anti-wrinkle, anti-acne, and skin-brightening (whitening) effects, spirulina has been used as a component of natural skin-care cosmetics as a moisturising and healing ingredient [4,7]. Microalgae rich in active metabolites have been used for the development of new drugs and healing-accelerating dressings, in tissue engineering, and as photosensitizers in photodynamic therapy [202]. Phycocyanin isolated from *S. platensis* and *S. maxima* has been used as an active pigment in natural hair dyes. Currently, there is a market demand for these pigments in the food, pharmaceutical, and cosmetic sectors [7]. Astaxanthin, lutein, β-carotene, and other active compounds contained in microalgae can be incorporated into dermo-cosmetics [12].

**Figure 5:**
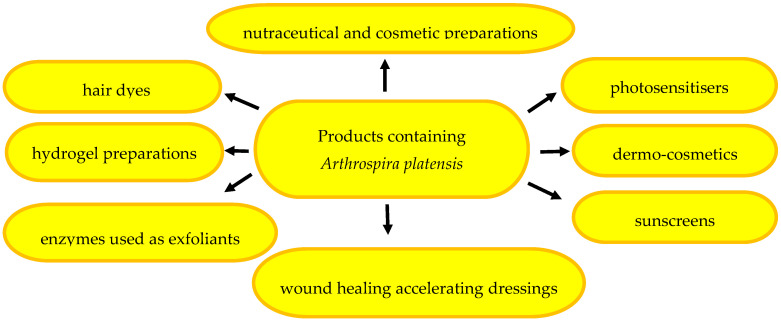
Application of *A. platensis* and *S. platensis* in selected pharmaceutical and cosmetic products.

Cyanobacterial biocompounds can be ingredients of products expanding the diversity of cosmetic formulations used as sunscreens and moisturisers in skin care and treatment of dermatological conditions [203]. Microalgal active metabolites contribute to the development of innovative products, e.g., *S. platensis* enzymes have been used as exfoliating agents to develop hydrogel preparations and cosmeceuticals [204]. With their properties, microalgae are promising components of the most modern natural cosmetics and cosmeceutical formulations based on environmentally friendly microalgal biomolecules replacing synthetic products [205]. The bioactive components of microalgae can serve as substitutes in the new trends in pharmaceutical, cosmetic, and cosmeceutical biotherapies [62,206]. In turn, the combination of spirulina and probiotics constitutes a new functional food strategy bringing positive effects on human health [207]. The application of *A. platensis* and *S. platensis* microalgae in various pharmaceutical and cosmetic products is presented in Figure 5.

### 2.6. Spirulina-Based Commercial Skin Care Products

There is no doubt that the diversity, quality, and topical types of *Spirulina* applications, as well as the availability and range of commercial Spirulina-based skin-care cosmetic products on the market, will rapidly expand in the following years.

In the cosmetic industry, there are commercially available skin protective and anti-aging active extracts from cyanobacteria, e.g., the spirulin extract Spiralin^®^ (Patents n° EP 2 563 478 B1 + US 2014/0127336 A1) used in such products as Skinicer^®^ Repair cream and Spirularin^®^, which stimulates fibroblast proliferation and inhibits hyaluronic acid digestion, providing regenerative effects on damaged skin cells and collagen and protection against UV radiation [208].

Bio-Botanica Company (USA) prepared a liquid blend of *Spirulina platensis* extract in glycerine and water offering skin conditioning benefits. In Italy, *Spirulina platensis* water extract stabilized by citric acid, sodium benzoate, and potassium sorbate with strong antioxidant potential is supplied by Phenbiox Company. Another active ingredient for skin application recommended for anti-aging cosmetic products is Spiruline AP^®^, which is a water-soluble extract of blue algae with excellent photoprotective, anti-inflammatory, antiradical, and cell-renewal effects designed by SEPPIC (France-headquarter). In turn, Sensient Cosmetic Technologies Company (Saint-Ouen-l’Aumône, France-headquarter) developed NatPure^®^ APX—a dry *Spirulina platensis* extract in dextrin, sodium phosphate, and sodium citrate with antioxidant potential, radiant skin effects, and revitalizing benefits [7].

In the currently marketed beauty products, *Spirulina platensis* and *S. maxima* are used as phycocyanin-rich blue extracts in exclusive formulations or as powder in cheaper products. There are two main spirulina skin care products containing *S. platensis* powder offered by puroBIO Cosmetics Company (Bari, Italy) and Ren Skin-care Company (London, UK). The former company sells the Kelly peel-off powder mask for dry skin, and the latter one produces a mattifying and purifying clay mask effective against blemishes; it removes sebum excess, minimises the appearance of pores, and fights congestion without drying the skin out. In turn, the relevant commercial cosmetic products based on *Spirulina maxima* extract include the *Spirulina* Santè Methode face line proposed by Santè Naturels Company (Milano, Italy), including the antioxidative anti-aging serum, restorative tonic, and balancing cleansing milk; Crème spiruline liftante Rider developed by Ella Bachè Nutridermologie (Paris, France) for winkle-lifting and anti-aging with progressive action; Marin Complex Deep Restorative Cream prepared by Zelens Company (London, UK), which restores and rejuvenates the skin without an irritation risk; the face and hand milk cream, SPF50+/30/15 after sun, hair oil, and tanning oil offered by Doni del Mare-Hanze Cosmetics (Milano, Italy) with moisturising, regenerating, and anti-aging properties; and the face serum and face cream developed by Institut Esthederm (Paris, France), providing immediate brightness effect and making the skin more toned, smooth, revitalised, and nourished. Two Australian companies, Helena Rubinstein and Sukin skincare, offer skin care products containing *S. platensis* extracts: Powercell Skinmunity Emulsion, which intensely hydrates the skin, smoothes wrinkles, and stimulates revitalisation; and face cream and face serum with nourishing, moisturising, antioxidant, revitalising, and detoxifying effects [7].

**Figure 6:**
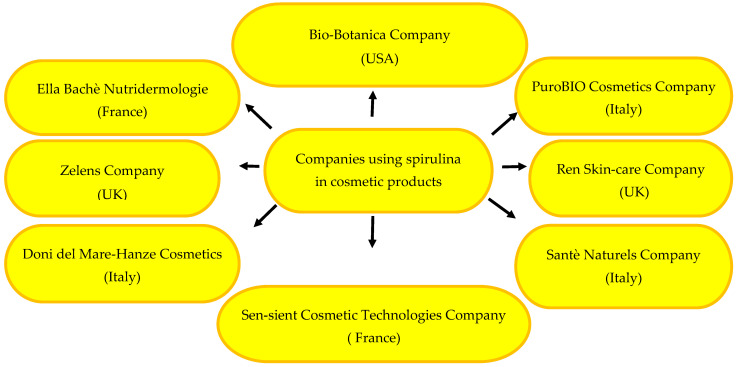
Companies using spirulina as an ingredient of various cosmetic products.

To sum up, a number of topical *Spirulina*-based formulations exhibit a large range of beneficial activities, e.g., revitalising, moisturising, antioxidant, remineralising, protecting, cleansing, and brightening effects. Therefore, these products may be a valuable component of skin-care formulations for treatment of skin pigmentation disorders, healthy sunscreen protection, and wound-healing benefits. The products can also be used topically as a booster of hydration, anti-acne, anti-aging, and anti-wrinkle action. Companies using spirulina derived from *A. platensis* and *S. platensis* to manufacture various cosmetic products are presented in Figure 6.

### 2.7. Strengths and Limitations

In topical skin treatment, the penetration of the drug through the skin is of key importance, which is the subject of many studies on the chemical nature of drugs and drug carriers [209,210]. As shown in this literature review, spirulina preparations do not require advanced techniques to facilitate their action on the skin, such as iontophoresis or microneedling [211,212]. Even without such techniques, the formulations exert therapeutic effects; nevertheless, special nanoparticles such as chitosan can enhance the therapeutic activity of spirulina [27,43,54,213]. The small number of side effects and their low frequency are advantages of the use of spirulina. This means that this drug can help treat acne and reduce the exposure to side effects of some other drugs (topical retinoids) [214]. The major problem is the difficulty in achieving microbiological purity of spirulina-containing formulations produced on an industrial scale and, consequently, the resulting risk of contamination with cyanobacterial toxins [189]. A more detailed description of the contraindications to the use of spirulina is presented in Section 2.4. Contraindications, while its advantages constitute the main part of this review.

Numerous limitations were indicated in studies on spirulina conducted by many researchers, e.g., the use of whole instead of purified spirulina extracts in analyses and the use of different cell systems, including other cells than human. Therefore, the interpretation of data on the potential use of spirulina in phytotherapeutic products requires detailed in vivo studies on human skin [215]. Inappropriate storage of spirulina affects its quality, and the technological process of spirulina biomass production causes the loss of some pro-health active chemical compounds [216]. In clinical practice, semi-solid formulations, i.e., creams, oils, ointments, and gels containing spirulina, are a limitation due to their insufficient skin penetration. This poses a challenge in developing new forms for topical application, including nanotechnology products, as nanofibres, microparticles, nanoparticles can enhance the treatment of skin diseases [146]. In various studies, a limitation was the small amount of spirulina given to patients, which ensures better cooperation of the organism and fewer undesirable side effects [217]. Another limitation is the decrease in the number of participants during the experiment and difficulties in the assessment of the effectiveness of topical application or supplementation as well as the insufficient application time to determine the effect. Hence, extension of the duration of experiments is recommended [218]. The limitations in spirulina supplementation included the impossibility of conducting subgroup analysis regarding gender or BMI, differences in health status, and a small number of participants in the study group [219]. Other limitations were the different types of spirulina supplements, different doses, variable time of use, and different lifestyles of participants during the experiment [91]. Despite the limitations, numerous studies have shown the effectiveness and potential clinical applications of spirulina e.g., in dermatological problems. *Arthospira platensis* and *Spirulina platensis* microalgae showed pro-health effects and alleviated the symptoms of skin diseases [7,147,202,217,220,221].

### 2.8. Future Research

It has been evidenced that aqueous extracts of algae with high levels of biological activity can be a source of antioxidants and pro-health nutrients [222]. Further research is required to facilitate the use of new biologically active compounds from microalgae as components of drugs and dietary supplements and to obtain high-quality biomass, also in terms of purity. A continuation of animal studies and clinical trials will determine new pharmacological activities and pharmaceutical and medical implementations [223]. The diverse range of bioactive metabolites contained in algae may gain great importance in the manufacture of cosmetic products due to their multidirectional effects on the skin. Algae may be a promising source of active compounds with pro-health properties for skin applications, an effective and inexpensive alternative to synthetic products, and ingredients of cosmeceuticals [224]. Further toxicological studies are necessary to assess the safety of spirulina products characterised by minimal contamination with harmful elements or toxic compounds that may cause negative effects in humans [225].

To sum up, further investigations are necessary to determine the pro-health suitability of microalgae, including spirulina, to exert multidirectional effects on human skin. There is a need for controlled clinical trials to assess the effects of spirulina on specific dermatological problems and confirm the clinical role of spirulina supplementation in combination with other medical therapies. The mechanisms and metabolic pathways of the impact of spirulina metabolites on cytokines, which are upregulated in autoimmune skin diseases, need to be elucidated. Equally crucial is the identification of microalgal bioactive metabolites and immunomodulators with the highest efficiency in the treatment of certain dermatological problems. It is important to carry out toxicological tests of microalgal extracts to confirm the purity of the raw material. Currently, most studies are conducted in in vitro and animal models, but the effectiveness of spirulina in dermatological applications should be confirmed in clinical studies, and the safety of using microalgae in accordance with applicable standards should be determined.

## 3. Material and Research Methods

### 3.1. Comparative Analysis of Selected Properties of Microalgae

The current knowledge of the pro-health effects of *Arthrospira* and *Spirulina* microalgae was analysed. The analyses were mainly focused on their phytotherapeutic antiviral, antibacterial, anti-acne, photoprotective, and healing effects. The chemical profile of these microalgae was characterised, taking into account the phytotherapeutic role of some chemical compounds contained in algal biomass. Contraindications and applications in skin care and treatment of dermatological problems were indicated. Additionally, prospects for further research were presented. The review of the original scientific publications covers the period of the last five years 2019–2023 and the current year.

In recent years, in the era of artificial intelligence technology, significant progress has been noted in the molecular and cellular research, tissue engineering, and various specialties, such as medical and cosmetic dermatology, aesthetic medicine, plasma and regenerative medicine, and plastic surgery. The review included the total number of publications of 3691, 81, and 16 archived in the PubMed database according to the selected keywords: (i) spirulina, (ii) spirulina, skin, and (iii) spirulina, dermatology, in relation to the analysed period (2019–2024), in which the number of publications was 1766, 49, and 10, respectively, which accounted for 45.4, 60.5, and 62.5% (Table 1). Therefore, in the review, the period 2019–2024 with the highest percentage of publications in relation to their total pool was selected for the comparative analysis of the latest scientific reports consistent with the discussed issues (Table 4).

### 3.2. Phrases and Scientific Databases

Original research publications were sought using interdisciplinary and specialised scientific databases: EBSCO, Google Scholar, ISI Web of Science, Medline, ProQuest Central, ProQuest SciTech Collection, PubMed, ScienceDirect, Scopus, Springer, Taylor & Francis, Web of Knowledge, Web of Science, and Wiley Online Library. The original scientific publications subjected to the comparative analysis mainly represent the fields of medical biology, biological sciences, medical sciences, health sciences, agriculture, and horticulture. The following keywords were used to search for relevant publications: *Arthrospira*, *Spirulina*, biologically active chemical compounds, photosynthetic pigments, protein, fats, carbohydrates, chlorophyll, flavonoids, carotene, phenolic acids, minerals, vitamins, phenolic compounds, contraindications, application, dermatological diseases, acne, phytotherapy, skin, and antiviral, antibacterial, anti-acne, photoprotective, and healing properties.

### 3.3. Number of Publications Found and Source Analysis Method

In the large collection of original scientific publications found in the aforementioned scientific databases, 225 thematically coherent scientific reports were selected and cited in this review, i.e., 61 publications were cited in the Introduction and 164 papers were cited in the Results section. The original research publications were screened for data on the phytotherapeutic properties of selected *Arthrospira* and *Spirulina* microalgae suitable for use in skin care, prophylaxis of dermatological diseases, and phytotherapy.

The comparative analysis of available research data was carried out following a scheme focused on the following parameters: (i) research model, (ii) application of microalgal products, extracts, or active chemical compounds, (iii) concentration used, (iv) dose, (v) duration of use, (vi) mechanism of action, (vii) size of the experimental group, and (viii) main conclusions regarding skin care and treatment of dermatological conditions.

### 3.4. Current Number of Citations of Analysed Publications

The number of citations of the analysed original research publications is presented in a table in this review. The publications are grouped according to subsequent years. The table presents the number of citations of the publications in each year and in the whole analysed period (Table 5).

## 4. Conclusions

The antiviral mechanism of action of microalgal active compounds, i.e., proteins, vitamins, polysaccharides, terpenoids, and calcium spirulan, involves the inhibition of viral replication and penetration into host cells and the enhancement of human resistance to viral infections. Pigments (mainly phycocyanins) isolated from *A. platensis* and *S. platensis* microalgae have a health-enhancing effect on the skin microflora. They inhibit the growth of *Aspergillus flavus*, *A. niger*, *C. albicans*, *E. coli*, *K. pneumoniae*, *Proteus vulgaris*, *Pseudomonas aeruginosa*, *Salmonella typhi*, *S. typhimurium*, *Serratia marcescens*, and *Staphylococcus aureus* and bacteria from the genus *Vibrio*. With their antibacterial and nutritional properties, microalgal active compounds can serve as ingredients in human skin care and therapy products.

Microalgal active compounds from the groups of alkaloids, alkanes, phenols, phycocyanins, tannins, saponins, steroids, alkenes, phthalates, phthalic acids, and carboxylic acids exhibit anti-acne activity against Gram-positive (*Aerococcus* spp., *Cutibacterium acnes*, *Enterococcus* spp., *P. acnes*, *S. aureus*, and *S. epidermidis*) and Gram-negative (*E. coli*, *Enterobacter aerogenes*, *P. aeruginosa*, and *Pseudomonas stutzeri*) bacteria. Novel formulations, i.e., spirulina nanoparticles with chitosan, as well as components of facial masks and creams, contribute to inhibition of tyrosinase activity, thus acting against resistant bacteria in acne therapy. The antibacterial activity is also influenced by the components and structure of the bacterial cell wall. Microalgal active compounds accelerate the wound healing process through the inhibition of inflammatory and apoptotic processes, stimulation of the proliferation of fibroblasts, keratinocytes, and polymorphonuclear cells, enhancement of the viability of cells supporting the granulation process, stimulation of angiogenesis and expression of proteins involved in the regeneration of damaged tissues, production of cytokines, an increase in the density of collagen fibres, and acceleration of proliferation of epithelial cells and formation of granulation tissue.

Innovative products containing pro-health metabolites in the form of nanophytosomes, encapsulated protein hydrolysates with nanoliposomes, and nanofibres accelerate the wound-healing process, strengthen antioxidant activity, and alleviate ROS stress, thereby contributing to increased rates of regeneration of skin wounds. The photoprotective properties of microalgae are mainly attributed to their content of amino acids, phlorotannins, carotenoids, mycosporins, and polyphenols. These phytochemicals exert a photoprotective effect via the inhibition of tyrosinase, secretion of pro-inflammatory cytokines, and generation of ROS in fibroblast cells and through enhancement of collagen production. Bilosomes loaded with microalgal metabolites are effective in the treatment of UV-induced skin damage. Microalgae are a source of promising molecular components of new parapharmaceuticals with various biomedical applications and many pharmacological and cosmetic formulations used in the prophylaxis and therapy of dermatological problems.

## Figures and Tables

**Table 1:** Antibacterial activity of *Spirulina platensis* in in vitro studies.

Spirulina/ Extract	Concentration/Mass	Bacterial Isolates	Main Conclusions	References
Acne–in vitro studies				
Methanol and hexane dry *S. platensis* extracts	100 µg in 100 µL H_2_O; concentrations: 25; 50, and 100 µL	*Micrococcus aureus*, *Propionibacterium acnes*, *Staphylococcus aureus*, and *S. epidermidis*, bacteria were isolated from the skin of acne patients (20 patients aged 15–20 years). Samples of bacterial isolates were prepared on nutrient agar with *S. platensis* extracts.	The hexane and methanol *S. platensis* extracts had antibacterial activity against *Aerococcus* spp. and *Enterococcus*. The following substances were involved in this activity: hexadecene, heptadecane, octadecene, 2-bromopropionic acid, methyl-1-docosene, benzenedicarboxylic acid, and tetradecanol.	[42]
Lyophilised *S. platensis* powder (SPP)	(i) 0.25% solution of *S. platensis* (SPP) in phosphate-buffered saline. (ii) *S. platensis*-containing creams with one of two different nonionic surfactants Tefose 63 (TFS) or sucrose ester SP 70 (SP70) incorporated in creams as emulsifying agents. (iii) Cream compositions (g): lyophilised *S. platensis* powder SPP (5), Transcutol HP (14.2), TFS or SP70 (3), cetostearyl alcohol (4.6), stearic acid (10), glycerol (5), IPM (5), propylene glycol (5), and purified water (ad 100).	*Staphylococcus aureus*, American Type Culture Collection (ATCC)^®^ 43300™) and *Cutibacterium* (formerly *Propionibacterium acnes* (ATCC^®^ 33169™)	*S. platensis* effectively reduced cell viability of *S. aureus* (66%) and *C. acne* (64%). *S. platensis* formulations with various surfactants, but especially the preparation containing the sucrose ester SP 70 emulsifying agent, were active against *C. acnes* and *S. aureus* comparably to Aknemycin™, and showed low toxicity on immortalized human keratinocyte Ha-CaT cells. Creams containing *S. platensis* can be an alternative option to treat acne with fewer side effects and without antibiotic resistance.	[57]
Anti-acne topical ointment of C-phycocyanin (C-PC) extracted from *S. platensis*	(i) formulation—oleaginous base (%): paraffin hard (5), wool fat (10), cetostearyl alcohol (10), white soft paraffin (50), liquid paraffin (15), extract C-PC (10), (ii) formulation—water base (%): PEG400 (12), PEG4000 (18), stearyl alcohol (28), extract C-PC (10), glycerine (17), and water q. s.	*P. acnes*, *S. epidermidis*	Spirulina has an anti-acne effect. Both of the topical C-PC ointment formulations can be employed in the treatment of acne against *P. acnes* and *S. epidermidis*. The formulation comprising the water-soluble base was superior to the oleaginous base due to the complete solubility of the extract in water.	[147]
Ethanolic *S. platensis* extract	Dried spirulina biomass was extracted with 96% ethanol using the reflux method and partitioned with hexane, distilled water, and ethyl acetate. The active compound fractions were analysed using thin layer chromatography and column chromatography.	*P. acnes*, *S. epidermidis*, and *Enterobacter aerogenes*	The highest activity was exhibited by ethyl acetate extracts against *P. acnes*, *S. epidermidis*, and *E. aerogenes* and ethanol extracts against *P. acne* and *S. epidermidis*. The ethyl acetate fraction of the *S. platensis* microalgae has the potential as a natural antibacterial agent.	[28]
Ethyl acetate and dimethyl carbonate *A. platensis* extracts; extract-loaded copper alginate-based nanocarriers	500 mg of *S. platensis* biomass, alginate-based nanocarriers (ANCs) were prepared using ultrasound oil-in-water emulsification followed by surface gelation with cupric ions.	*Cutibacterium acnes* ATCC^®^ 6919	*A. platensis* extracts prevented the growth of *C. acnes* single-species biofilms (inhibition > 75% at 0.2 mg/mL). Nanovectorised extracts reduced the growth of both single-species (inhibition > 43% at 0.2 mg/mL) and preformed (55–77%) *C. albicans* ATCC^®^ 28367™ biofilms	[153]

Explanation: quantum satis (q. s); minimal inhibitory concentration (MIC), registered (^®^), trademark (TM).

**Table 2:** Healing effect of selected microalgae in animal studies.

Spirulina/Extract	Research Model	Animal Age	Number of Experimental Groups	Number of Animals	Duration of EXPERIMENTS (Days)	Main Conclusions	References
Healing effects							
Spirulina protein (SPCP)	C57BL/6 mice	n.d.	1G. Control 2G. Treated with Vaseline containing 10 μg/g of epidermal growth factor (EGF) 3G. Treated with Vaseline containing 2% of SPCP 4G Treated with Vaseline containing 4% of SPCP and EGF	20	5	SPCP proved to be an effective phytotherapeutic ingredient supporting wound healing. The ERK, Akt, and TGF β1 signalling pathways played a major role in this process.	[46]
Spirulina ethanol extract	Wistar rats	2–3 months	1G. Negative control—physiological saline solution 2G. Positive control—0.1% Gentamicin ointment 3G. *S. platensis* extract and 0.1% cream 4G. *S. platensis* extract and 0.1% ointment	30	14	Ointments with *S. platensis* extract (0.1%) increased the number of fibroblasts and accelerated the wound healing process.	[168]
Phycocyanin *S. platensis*	*Candida*-infected mice	10 weeks	1G. Control treated with cream with no active substance 2G. Treated with cream with 1.5% of phycocyanin 1G. Treated with cream with 3% of phycocyanin	15	14	The wound size was calculated using an appropriate equation.	[173]
Spirulina (5%)	Wistar rats	3–4 months	1G. Control 2G. Diabetic rats receiving standard diet 3G. Diabetic rats with spirulina supplementation (50 g/kg/day) 4G. Diabetic rats with chlorella supplementation (50 g/kg/day) 5G. Diabetic rats with chlorella and spirulina supplementation (25 g/kg/day of chlorella and 25 g/kg/day of spirulina)	65	21	The diet of diabetic rats supplemented with spirulina, chlorella, and their combination had a beneficial effect on wound healing, e.g., it improved the formation of granulation tissue, vascularisation, and regeneration of epithelium tissue. Spirulina and chlorella were recommended for use in phytotherapy of various types of wounds.	[174]
*S. platensis* water extract	‘Swiss Albino’ mice	8–10 weeks	1G. Control 2G. Treatment with 125 mg/kg of biafine ointment (trolamine 0.67 g/100 g) 3G. Aqueous solution of spirulina paste (0.5%)	n.d.	35	The extract accelerated the healing of mechanical, chemical, and thermal burns and hair growth. Spirulina can be used as a therapeutic wound healing agent in complementary therapy and conventional medicine.	[175]
	‘White’ rabbits	2–3 months		9			

Explanation: no data (n.d); group (G).

**Table 3:** Pro-health effects of some microalgae in clinical trials.

Spirulina/Extracts	Concentration/Mass	Number of Patients	Patient Age (Years)	Application	Duration of Application	Main Conclusions	References
Pro-health-promoting effects on the skin—clinical trials							
Spirulina extract	0.1%	25 males 25 females	18–65	twice a day	1 year	Spirulina in skin care strengthened the skin barrier and exhibited moisturising and anti-aging properties.	[60]
Gel-cream formula (carrier—FGV with 0.1% spirulina extract (FGA)	0.1% extract	50 females 50 males	18–65	twice a day	1 year	The treatment resulted in an increase in water content in the *stratum corneum*, a reduction in TEWL, a significant reduction in sebum content, improvement in skin microrelief via reduction in surface roughness, and more even distribution and homogeneity of keratinocytes.	[60]
Scalp and hair—clinical trials							
*S. platensis* and *Ascophyllum nodosum* dry extract	Dry extract of 0.1% of *S. platensis* and 2% of *A. nodosum*	26	18–35	twice a day	n.d	The treatment resulted in a decrease in sebum content, combing force, and improved mechanical properties and hair gloss. Spirulina can be a beneficial ingredient of an innovative hair-conditioner recipe.	[186]

**Table 4:** Number of publications according to selected keywords in accordance with the PUBMED database (access date 19 September 2024).

Keywords	Years		Number of Publications	Percentage of Publications
Spirulina	archived in the database	1967–2024	3691	100
	analysed in the review	2019–2024	1766	45.41
Spirulina, skin	archived in the database	1981–2024	81	100
	analysed in the review	2019–2024	49	60.5
Spirulina, dermatology	archived in the database	2004–2024	16	100
	analysed in the review	2019–2024	10	62.5

**Table 5:** Number of original scientific publications in each year and number of citations of the articles according to the Google Scholar database on 23 September 2024.

Year of Publication	Number of Publications in Each Year	Bibliographic Number of Cited Publications	Number of Citations	Total Number of Citations in Each Year
2019	27	[9,16,17,21,51,53,58,69,83,88,90, 94,106,110,118,141,142,143,149,169,171, 172,177,184,186,218,225]	79, 142, 13, 1, 34, 20, 12, 140, 44, 27, 11, 148, 54, 13, 60, nc, 69, 27, 6, 17, 33, 1, 115, 11, 7, 72, 95	1251
2020	40	[1,13,26,30,38,46,52,55,57,62,68, 75,77,81,84,86,96,111,112,115,121, 130,140,145,148,150,153,167,168,170,173, 175,176,181,192,204,207,208,214,224]	6, 4, 4, 37, 3, 13, 21, 1, 13, 124, 2, 31, 28, 2, 93, 25, 20, 1, nc, 21, 13, 2, 2, 4, 11, 408, 8, 2, 2, 28, 9, 5, 35, 3, 6, 93, 5, 44, 44, 143	1314
2021	37	[2,7,8,12,22,27,39,40,42,44,47, 60,79,87,91,92,93,95,100,103,119, 122,123,127,128,131,133,134,138,151,156, 174,189,190,195,211,219]	58, 64, 1, 8, 40, 51, 24, 57, 2, 90, 10, nc, 45, 25, 16, 73, 43, 31, 32, 16, 18, 5, 57, 51, 2, 11, 92, 25, 1, nc, 51, 11, 18, 14, 4, 138, 24	1208
2022	57	[3,11,14,15,18,23,28,32,34,35,43, 49,56,59,63,70,71,73,74,76,85,89, 97,98,101,102,104,107,108,113,120,124, 125,129,136,137,139,146,147,154,155,161, 163,178,179,193,196,198,200,205,206,212, 216,217,220,223]	11, 22, nc, 1, 92, 38, 9, 11, 5, 96, 8, 19, 2, 2, 55, 33, 71, 32, 49, 15, 6, 2, 129, nc, 23, 13, 3, 23, 2, 5, nc, 1, 4, 10, 14, 2, nc, 16, nc, 1, 8, 15, 48, 3, 4, 3, 2, 40, 4, 9, 50, 81, 7, 10, nc, 22, 3	1134
2023	44	[4,5,6,10,19,24,25,29,31,33,36, 37,45,48,50,64,65,75,78,82,99,105, 114,116,126,135,144,157,159,160,162,164, 165,166,180,182,187,188,191,194,199,202, 209,213]	7, 18, 2, 12, 31, 1, 7, 8, 2, 5, 5, 13, 21, 5, 10, 35, 2, 12, 4, 37, 4, 7, 7, 10, nc, 9, 65, 2, 8, nc, 3, nc, 2, 4, 3, nc, 28, 3, 4, 1, 2, 16,4, 106	525
2024	20	[20,41,54,61,66,67,80,109,117,132,152, 158,183,185,197,201,210,215,217,221,222]	4, 2, 1, 1, 1, 2, nc, nc, 2, 4, nc, 5, 1, 3, 1, 3, nc, nc, nc, 9, nc	39

Explanations: for the consecutive bibliographic number given in the third column, the corresponding number of citations is given in the fourth column; no citation—nc.
